# Age, Motion, Medical, and Psychiatric Associations With Incidental Findings in Brain MRI

**DOI:** 10.1001/jamanetworkopen.2023.55901

**Published:** 2024-02-13

**Authors:** Russell H. Tobe, Lucia Tu, Maya Roberts, Gregory Kiar, Melissa M. Breland, Yiwen Tian, Minji Kang, Rachel Ross, Margaret M. Ryan, Emmanuel Valenza, Lindsay Alexander, Anna MacKay-Brandt, Stanley J. Colcombe, Alexandre R. Franco, Michael P. Milham

**Affiliations:** 1 Nathan S. Kline Institute for Psychiatric Research, Orangeburg, New York; 2 Center for the Developing Brain, Child Mind Institute, New York, New York; 3 Center for Data Analytics, Innovation, and Rigor, Child Mind Institute, New York, New York; 4 Infervision, Philadelphia, Pennsylvania; 5 St John’s University, Staten Island, New York; 6 Icahn School of Medicine at Mount Sinai, New York, New York; 7 Department of Psychiatry, New York University Grossman School of Medicine, New York

## Abstract

**Importance:**

Few investigations have evaluated rates of brain-based magnetic resonance imaging (MRI) incidental findings (IFs) in large lifespan samples, their stability over time, or their associations with health outcomes.

**Objectives:**

To examine rates of brain-based IFs across the lifespan, their persistence, and their associations with phenotypic indicators of behavior, cognition, and health; to compare quantified motion with radiologist-reported motion and evaluate its associations with IF rates; and to explore IF consistency across multiple visits.

**Design, Setting, and Participants:**

This cross-sectional study included participants from the Nathan Kline Institute–Rockland Sample (NKI-RS), a lifespan community-ascertained sample, and the Healthy Brain Network (HBN), a cross-sectional community self-referred pediatric sample focused on mental health and learning disorders. The NKI-RS enrolled participants (ages 6-85 years) between March 2012 and March 2020 and had longitudinal participants followed up for as long as 4 years. The HBN enrolled participants (ages 5-21 years) between August 2015 and October 2021. Clinical neuroradiology MRI reports were coded for radiologist-reported motion as well as presence, type, and clinical urgency (category 1, no abnormal findings; 2, no referral recommended; 3, consider referral; and 4, immediate referral) of IFs. MRI reports were coded from June to October 2021. Data were analyzed from November 2021 to February 2023.

**Main Outcomes and Measures:**

Rates and type of IFs by demographic characteristics, health phenotyping, and motion artifacts; longitudinal stability of IFs; and Euler number in projecting radiologist-reported motion.

**Results:**

A total of 1300 NKI-RS participants (781 [60.1%] female; mean [SD] age, 38.9 [21.8] years) and 2772 HBN participants (976 [35.2%] female; mean [SD] age, 10.0 [3.5] years) had health phenotyping and neuroradiology-reviewed MRI scans. IFs were common, with 284 of 2956 children (9.6%) and 608 of 1107 adults (54.9%) having IFs, but rarely of clinical concern (category 1: NKI-RS, 619 [47.6%]; HBN, 2561 [92.4%]; category 2: NKI-RS, 647 [49.8%]; HBN, 178 [6.4%]; category 3: NKI-RS, 79 [6.1%]; HBN, 30 [1.1%]; category 4: NKI-RS: 12 [0.9%]; HBN, 6 [0.2%]). Overall, 46 children (1.6%) and 79 adults (7.1%) required referral for their IFs. IF frequency increased with age. Elevated blood pressure and BMI were associated with increased T2 hyperintensities and age-related cortical atrophy. Radiologist-reported motion aligned with Euler-quantified motion, but neither were associated with IF rates.

**Conclusions and Relevance:**

In this cross-sectional study, IFs were common, particularly with increasing age, although rarely clinically significant. While T2 hyperintensity and age-related cortical atrophy were associated with BMI and blood pressure, IFs were not associated with other behavioral, cognitive, and health phenotyping. Motion may not limit clinical IF detection.

## Key Points

**Question:**

What are the prevalence and health associations of incidental findings (IFs) in brain magnetic resonance imaging (MRI)?

**Findings:**

In this cross-sectional study of 4072 participants (ages 5-85 years), 9.6% of children and 54.9% of adults had any IF; 1.6% of children and 7.1% of adults required referral for these findings. Although cortical atrophy and T2 hyperintensities were associated with higher body mass index in adults, IFs had no other health associations.

**Meaning:**

In this study, IFs were common and rarely of clinical concern; however, cortical atrophy and/or T2 hyperintensities may warrant critical consideration.

## Teaser

This cross-sectional study examines rates of brain-based incidental findings on brain magnetic resonance imaging (MRI), their persistence, and their associations with phenotypic indicators of behavior, cognition, and health among US adults and children.

## Introduction

Incidental findings (IFs) in brain magnetic resonance imaging (MRI) of otherwise neurologically and medically asymptomatic populations are common and range from normal anatomic variants to severe illness. Children typically have lower rates of clinically significant IFs, at approximately 1% to 4%,^1,2,3^ while rates in older adults may be as high as 10%.^4^ However, there is no accepted convention for reporting IFs, which limits comparison between investigations. While the importance of detecting low-incidence high-acuity IFs (eg, neoplasm) is widely accepted, the clinical associations of common low-acuity findings (eg, T2 hyperintensities) have been incompletely evaluated. This is likely due to limited sample size; age ranges; and neurocognitive, psychiatric, and health phenotyping.

Research participants and clinical patients with low-acuity IFs often pursue additional clinical evaluations and scans to clarify findings or monitor temporal stability. These require allocation of limited medical services while imparting potential financial and emotional strain. Common IFs that tend to both be stable longitudinally and rarely have cognitive, behavior, or physical health associations may not warrant further clinical surveillance or evaluation. Therefore, there is an unmet need to explore whether common IFs are associated with other health indices in a manner that potentially guides clinical management in addition to identifying stability in their detection over time.

Participant motion during scans may impact interpretability by introducing artifacts.^5,6,7,8^ Children,^6,7^ males,^7^ and certain clinical populations^7,9^ tend to have more motion. Given the implications in data quality, quantitative motion characterization strategies such as the Euler number^10^ are increasingly deployed in research settings, although the associations of these quantitative strategies with radiologist-reported motion artifacts and their relevance with respect to IF detection are incompletely understood. Euler numbers are calculated in Freesurfer. Participants with higher degrees of motion or other artifacts will have a greater Euler number, which corresponds with the number of holes in cortical meshes and acts as a measure of quality. Prior work has shown a high correlation between Euler number and manual motion ratings.^10,11,12^

The Nathan Kline Institute–Rockland Sample (NKI-RS)^12,13^ and Healthy Brain Network (HBN)^14^ are large neuroimaging cohorts with robust cognitive, physical, behavioral, and psychiatric phenotyping. While not formally harmonized, they share several phenotypic characterizations and utilize a common clinical neuroradiology core to review scans. The initiatives differ in several ways: (1) NKI-RS enrolls participants living in the New York–New Jersey metropolitan division, while HBN predominantly enrolls participants from New York City; (2) enrollment in NKI-RS includes individuals aged 6 to 85 years, while HBN includes those aged 5 to 21 years; (3) NKI-RS is community-ascertained, while HBN is a community self-referred sample focused on mental health and learning disorders; and (4) NKI-RS follows some participants longitudinally.

In this study, we add to prior efforts by reporting overall rates of brain-based IFs across the lifespan and evaluating their temporal persistence while exploring associations with other phenotypic indicators of health, behavior, and cognition in the hopes this will lend novel insight into the clinical associations of such findings. We additionally investigate the associations between Euler number and radiologist-reported motion artifacts to (1) see whether automated quantified motion approaches can supplement clinical interpretation and (2) determine associations between general motion and IF detection rates. Finally, we evaluate the consistency, or recall, of IF detection over time, which may have relevance in clinical monitoring of IFs.

## Methods

### Participants

Research scans were reviewed by the clinical neuroradiology core for 1300 NKI-RS participants aged 6 to 85 years enrolled from March 2012 to March 2020 and 2772 HBN participants aged 5 to 21 years enrolled from August 2015 to October 2021. The NKI-RS had longitudinal participants followed up for as long as 2.5 years (enrollment ages, 6-17 years) or 4 years (enrollment ages, 38-71 years). Participants lacking assent or consent capacity or demonstrating cognitive, medical, or neurologic impairments limiting protocol engagement were excluded. Participants with autism spectrum disorder, history of suicidality, or history of psychiatric hospitalization were eligible for the HBN but not the NKI-RS. The NKI-RS and the HBN were reviewed and approved by the NKI institutional review board and the Advarra institutional review board, respectively. Adult participants provided written consent. Child participants and parents or legal guardians provided written assent and written consent, respectively. This study follows the Strengthening the Reporting of Observational Studies in Epidemiology (STROBE) reporting guidelines for cross-sectional studies.

### Measures

The NKI-RS and HBN maintain several joint measures (eMethods in Supplement 1) among the shared age range of the initiatives, including the Kiddie-Schedule for Affective Disorders and Schizophrenia (KSADS)^15^; the Child Behavior Checklist (CBCL)^16^; and the Wechsler Abbreviated Scale of Intelligence–Second Edition (WASI-II).^17^ For adults in the NKI-RS, the Structured Clinical Interview for *DSM-IV* Disorders (SCID-IV)^18^ and the Adult Self Report (ASR)^19^ were used in place of the KSADS and CBCL, respectively. NKI-RS adults also completed the Quick Screen adapted by National Institute on Drug Abuse (NIDA)^20^ and NIDA-modified Alcohol, Smoking, and Substance Involvement Screening Test (ASSIST).^21^

Toward the goal of detailed sample characterization, demographic information was reported by parents or legal guardians for participants ages 6 to 11 years and self-reported by participants ages 12 years and older. The Office of Management and Budget–required race categories (ie, American Indian and Alaska Native, Asian, Black or African American, Native Hawaiian and Other Pacific Islander, and White) were used.

### MRI and Reporting

Detailed information regarding imaging protocols, scanner type, and specifications can be found in Tobe et al,^12^ Alexander et al,^14^ and eMethods in Supplement 1. While site-specific differences in teams (including radiology technicians) may have impacted motion, both scanners produced clinically adequate structural scans for neuroradiologist interpretation that were reviewed by an independent neuroradiology group shared between both initiatives (see the eMethods in Supplement 1 for further details). The neuroradiologists issued written reports outlining clinical findings, presence of clinically significant motion, and follow-up recommendations. For longitudinal NKI-RS participants, radiologists could reference past scans and reports.

### MRI Report Coding

Approximately 196 distinct IF types were clinically reported in both samples (eTable 1 in Supplement 1). Similar IFs were grouped (eTable 2 in Supplement 1), yielding 66 final IF categories, of which 42 were considered brain-based (eTable 3 in Supplement 1). Multiple individual IFs within a single radiology report were coded independently. The team adapted the Adolescent Brain Cognitive Development Consortium group coding convention.^1,2,3^ Each IF was assigned a significance of 1 for no abnormal findings; 2, normal anatomic variant or common IF unlikely to be of clinical significance, no referral recommended; 3, consider referral; or 4, consider immediate referral. Radiologist-reported motion artifacts were coded as either present (reported) or absent.

Neuroradiologist-issued MRI reports were coded by trained research staff on each protocol (M.M.R. and L.T. for the NKI-RS; R.R. and E.V. for the HBN) from June to October 2021. The teams met jointly with a study physician (R.H.T.) for weekly coding meetings to clarify coding questions, discuss coder discrepancies, and determine groupings of IF types. R.H.T. reviewed all category 3 and 4 findings to confirm appropriate coding.

### Interrater Reliability

NKI reviewers independently corated a random subset of 517 MRI reports. The NKI-RS was selected given the higher rates of IF occurrences due to inclusion of adults.

### Statistical Analysis

Data were analyzed from November 2021 to February 2023 using Python version 3.11 (Python Software Foundation). All analyses used α = .05 to determine statistical significance. Only brain-based IFs were included for analyses (eTable 3 in Supplement 1). To compare rates between population groups or binary variables, we used proportion tests of 2 independent samples: 2-sided tests to assess associations between presence of motion artifacts and IF rates and 1-sided tests to assess whether the presence of psychiatric diagnoses was associated with increased IF rates. To compare the distribution of continuous variables for participants with and without brain-based IFs, we used 1-sided Mann-Whitney *U* tests. When drawing a conclusion across multiple statistical tests, we corrected *P* values for multiple comparisons using the Holm-Bonferroni method. To create a projection model, we used confound-isolating cross-validation^22^—a sampling approach that creates a test set in which the outcome of interest is independent from a confounding factor—to account for the confounding effect of age on the prevalence of IFs in our training data. Given the age associations with instances of T2 hyperintensities, we trained a random forest classifier as a post hoc analysis to determine which physiologic variables, if any, among blood pressure (diastolic or systolic), body mass index (BMI; calculated as weight in kilograms divided by height in meters squared), waist circumference, weight, and sex were most associated with the reporting of T2 hyperintensity. We selected a random forest model here, as it is a nonparametric approach that provides a highly interpretable summary of the importance of each feature in a projection relative to one another.

## Results

### Participants

A total of 4072 participants had clinical neuroradiology MRI reports. There were 1300 NKI-RS participants (781 [60.1%] female; mean [SD] age, 38.9 [21.8] years) and 2772 HBN participants (976 [35.2%] female; mean [SD] age, 10.0 [3.5] years).

### IF Prevalence and Demographic Descriptors

**Figure 1.**
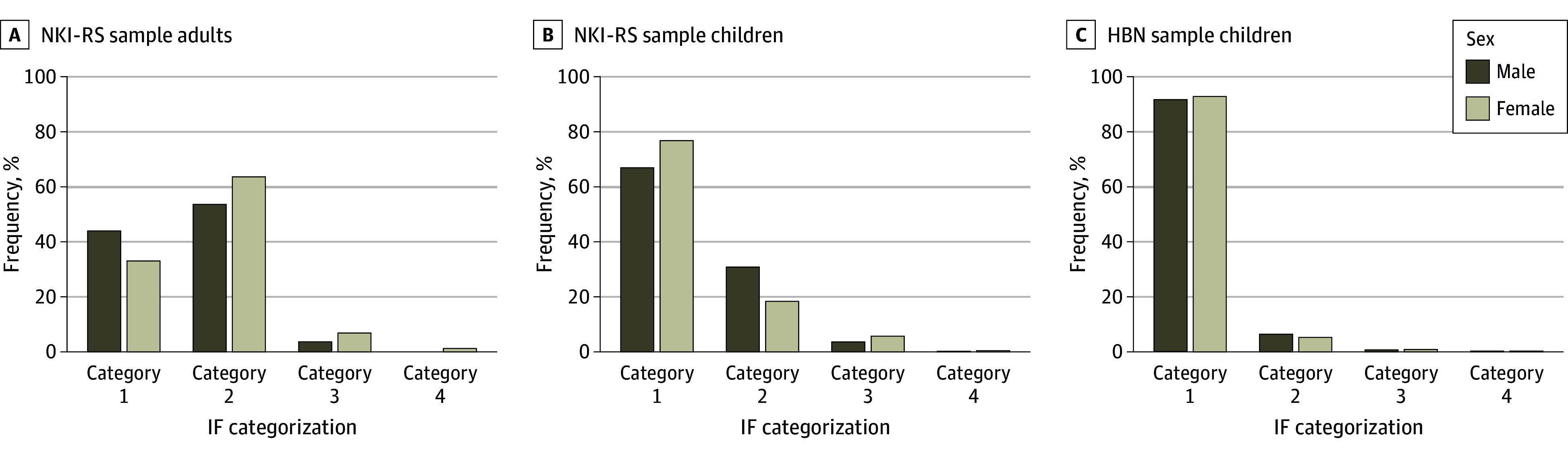
Incidental Finding (IF) Rates by Birth Sex Rates of IFs for birth sex are displayed for Nathan Kline Institute–Rockland Sample (NKI-RS) adults (ages ≥22 years) (A), NKI-RS children (ages 6-21 years) (B), and Healthy Brain Network (HBN) children (ages 6-21 years).

Descriptive sample characteristics including demographic and income information stratified by IF categories are presented in the Table. IFs were commonly reported but rarely of clinical concern (NKI-RS: category 1, 619 [47.6%]; category 2, 647 [49.8%]; category 3, 79 [6.1%]; category 4, 12 [0.9%]; HBN: category 1, 2561 [92.4%]; category 2, 178 [6.4%]; category 3, 30 [1.1%]; category 4, 6 [0.2%]) (Table). Among participants in the shared age range between the protocols (ages 6-21 years), rates of IFs were lower in the HBN (202 [7.8%]) compared with the NKI-RS (114 [28.8%]) (*P* < .001). This was driven by a significant difference in category 2 rates (HBN, 170 [6.6%]; NKI-RS, 103 [26.0%]; *P* < .001) rather than findings warranting clinical follow-up (Figure 1).

**Table.** Prevalence of Incidental Findings and Demographic Data in 1300 Participants in NKI-RS and 2772 in HBN

Variable	Participants, No. (%)^a^							
	Category 1		Category 2		Category 3		Category 4	
	NKI-RS	HBN	NKI-RS	HBN	NKI-RS	HBN	NKI-RS	HBN
Total^b^	619 (47.6)	2561 (92.4)	647 (49.8)	178 (6.4)	79 (6.1)	30 (1.1)	12 (0.9)	6 (0.2)
Sex								
Males	271 (43.8)	1621 (63.3)	227 (35.1)	120 (67.4)	21 (26.6)	18 (60.0)	2 (16.7)	4 (66.7)
Females	348 (56.2)	940 (36.7)	420 (64.9)	58 (32.6)	58 (73.4)	12 (40.0)	10 (83.3)	2 (33.3)
Age, mean (SD), y	28.2 (16.9)	9.9 (3.4)	50.7 (20.7)	10.0 (3.8)	44.3 (21.2)	11.3 (3.9)	48.1 (23.4)	13.0 (4.7)
Race^c^								
American Indian and Alaska Native	6 (1.0)	4 (0.2)	6 (0.9)	0	2 (2.6)	0	0	0
Asian	42 (6.9)	71 (3.4)	27 (4.2)	7 (4.7)	3 (3.8)	1 (4.5)	0	0
Black or African American	124 (20.3)	368 (17.8)	85 (13.2)	19 (12.8)	8 (10.3)	5 (22.7)	1 (8.3)	1 (25.0)
Native Hawaiian and Other Pacific Islander	4 (0.7)	2 (0.1)	0	0	1 (1.3)	0	0	0
White	407 (66.7)	1179 (57.0)	512 (79.5)	89 (60.1)	63 (80.8)	13 (59.1)	11 (91.7)	3 (75.0)
Some other race	27 (4.4)	36 (1.7)	14 (2.2)	6 (4.1)	4 (5.1)	0	0	0
≥2 Races^d^	NA	410 (19.8)	NA	27 (18.2)	NA	3 (13.6)	NA	0
Ethnicity^c^								
Hispanic or Latinx	89 (14.6)	591 (26.8)	61 (9.5)	34 (22.4)	5 (6.4)	2 (7.7)	2 (16.7)	1 (25.0)
Household annual income, $^e^								
<50 000	137 (22.1)	341 (13.3)	136 (21.0)	24 (13.5)	14 (17.7)	2 (6.7)	4 (33.3)	1 (16.7)
≥$50 000 to <$100 000	138 (22.3)	426 (16.6)	165 (25.5)	35 (19.7)	23 (29.1)	4 (13.3)	2 (16.7)	0
≥$100 000	152 (24.6)	1140 (44.5)	182 (28.1)	77 (43.3)	18 (22.8)	11 (36.7)	5 (41.7)	2 (33.7)

Abbreviations: HBN, Healthy Brain Network; NA, not applicable; NKI-RS, Nathan Kline Institute–Rockland Sample.

^a^ Category 1 indicates no abnormal findings; category 2, normal anatomic variant or common incidental finding unlikely to be of clinical significance in a healthy individual, no referral recommended; category 3, consider referral; category 4, consider immediate referral.

^b^ The total number of participants with each category classification are represented along with total percentages of participants in the respective sample. Birth sex and age distributions are listed as the total number of participants by category for each study with percentage of participants within that category classification. The total number of participants reporting race, ethnicity, and household annual income are listed by study in footnotes c and e. For race, ethnicity, and household income, the number of incidental finding (IF) instances for each category and percentage of the total IF instances within that category for all participants (regardless of reporting race, ethnicity, or household income) are listed by study. Of note, participants with multiple IFs can be represented in multiple category classifications. Therefore, percentages for a given row may not add up to 100.0% as individuals with multiple incidental findings across multiple categories will appear to be double counted from the perspective of individual demographic variables.

^c^ Overall, 1288 NKI-RS participants and 2243 HBN participants reported race; 1289 NKI-RS participants and 2388 HBN participants reported ethnicity. HBN and NKI-RS participants had 6 options when reporting race: American Indian and Alaska Native, Asian, Black or African American, Native Hawaiian and Other Pacific Islander, White, or some other race.

^d^ HBN participants had the option of identifying as more than 1 race. NKI-RS participants did not.

^e^ Overall, 930 NKI-RS participants and 2061 HBN participants reported income.

In the NKI-RS, older participants were more likely to have an IF, regardless of clinical significance (mean [SD] age: category 1, 28.2 [16.9] years; category 2, 50.7 [20.7] years; category 3, 44.3 [21.2] years; category 4, 48.1 [23.4] years). When comparing adults vs children directly, rates were significantly higher in adults for category 2 IFs (adults, 536 [60.7%]; children, 97 [25.1%]; *P* < .001) but not category 3 (adults, 55 [6.2%]; children, 19 [4.9%]; *P* = .40) or category 4 (adults, 10 [1.1%]; children, 2 [0.5%]; *P* = .40) IFs. There was no significant association of birth sex or household income with IF rates after controlling for age. Most frequently reported IFs for each category in the NKI-RS and HBN are listed in eTable 4 in Supplement 1.

**Figure 2.**
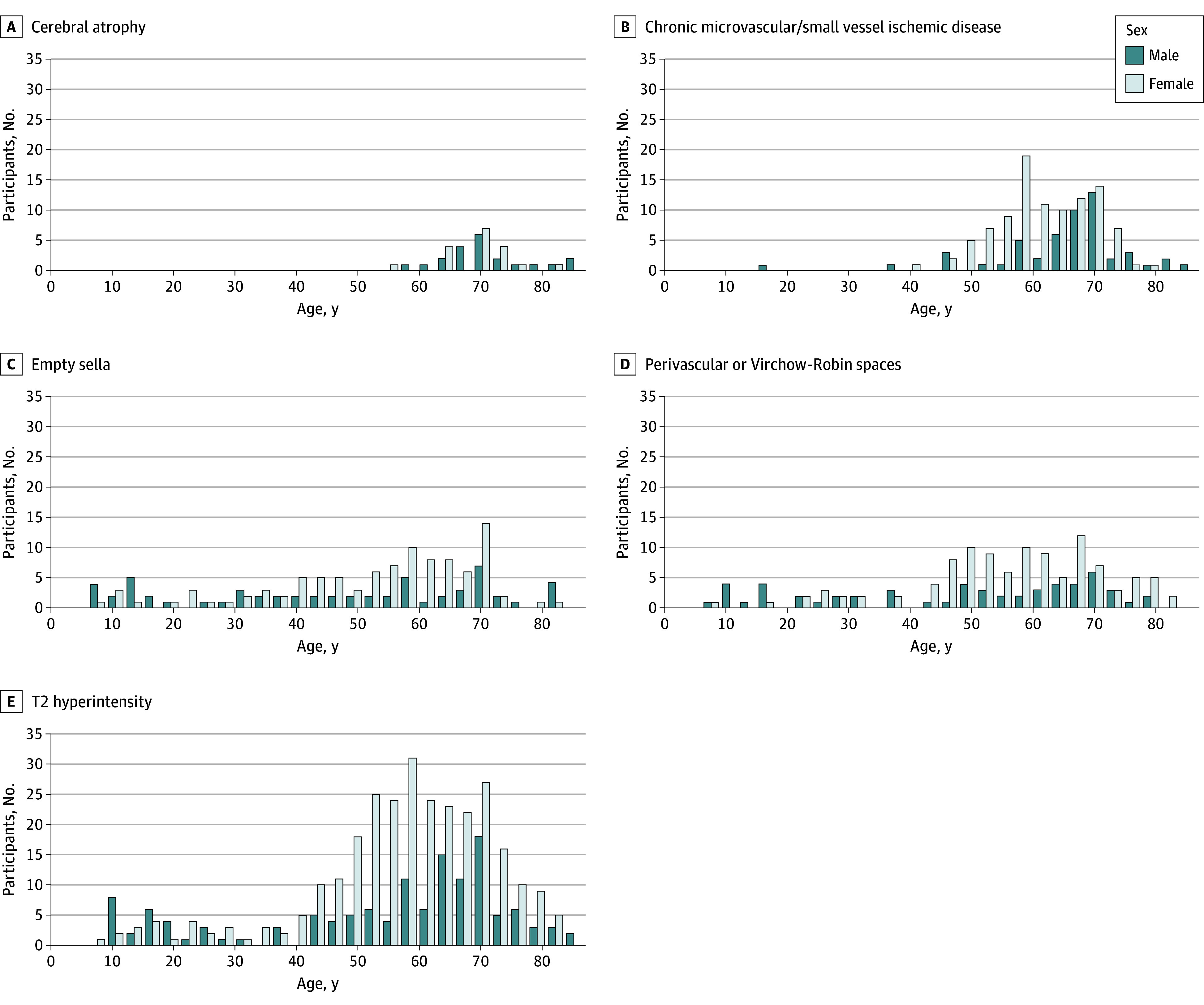
Frequency of Common Incidental Findings by Age in the Nathan Kline Institute–Rockland Sample Frequency of incidental findings present in more than 25 participants in the Nathan Kline Institute–Rockland Sample are represented.

Types of category 2 and 3 IFs varied by age. The merged sample between the NKI-RS and HBN demonstrated 284 of 2956 children (9.6%; ages 5-17 years; mean [SD] age, 9.9 [3.2] years) and 608 of 1107 adults (54.9%; ages 18-85 years; mean [SD] age, 45.4 [18.5] years) had at least 1 IF. For children, 46 (1.6%) required referral and 7 (0.2%) urgent referral. For adults, 79 (7.1%) required referral and 11 (1.0%) urgent referral. Among 11 IFs reported at least 25 times in the NKI-RS sample, cerebral atrophy (mean age with finding, 71.0; mean age without finding, 37.7; *P* < .001), chronic microvascular disease (mean age with finding, 62.4; mean age without finding, 35.2; *P* < .001), empty sella (mean age with finding, 48.9; mean age without finding, 37.3; *P* < .001), perivascular spaces (mean age with finding, 53.4; mean age without finding, 37.4; *P* < .001), and T2 hyperintensity (mean age with finding, 55.8; mean age without finding, 29.6; *P* < .001) were statistically correlated associated with age (Figure 2), while pineal cysts (mean age with finding, 38.7; mean age without finding, 38.5; *P* > .99) and cerebellar tonsillar ectopia (mean age with finding, 29.1; mean age without finding, 38.3; *P* > .99) were not. For HBN, no IFs had a significant association with age.

### Associations of IF With Psychiatric and Physical Health Phenotyping

**Figure 3.**
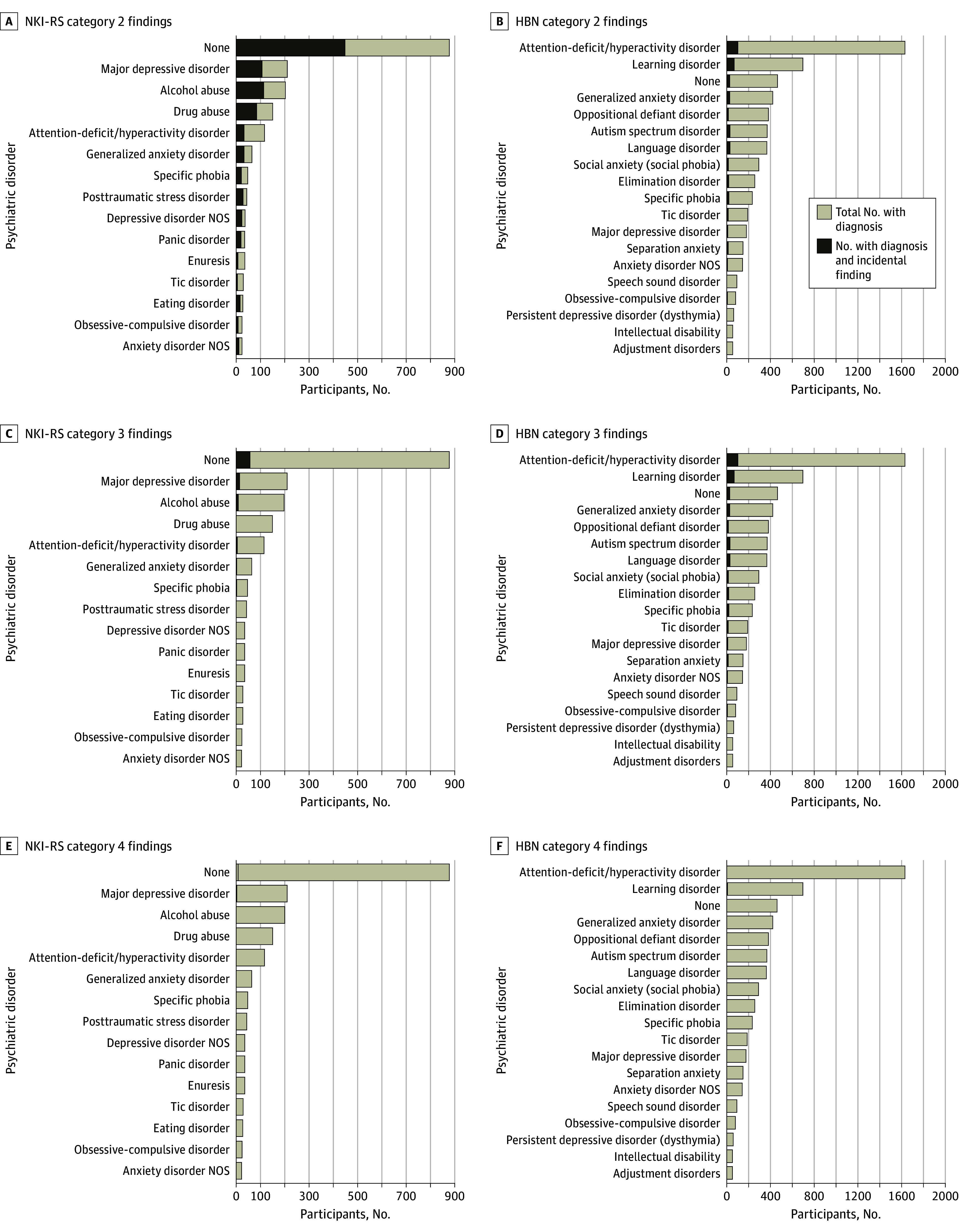
Frequency of Incidental Finding Severity by Psychiatric Diagnosis Frequency of incidental finding severity by psychiatric diagnosis is represented for the Nathan Kline Institute–Rockland Sample (NKI-RS) and the Healthy Brain Network (HBN). The count of participants with findings overlays the count of participants with psychiatric diagnosis. NOS indicates not otherwise specified.

Rates of IFs by presence or absence of categorical psychiatric diagnoses are represented in Figure 3. Risk ratios for psychiatric diagnoses are in eTable 5, eTable 6, and eTable 7 in Supplement 1. No psychiatric diagnoses were associated with any IF or IF severity after accounting for age. There also was no significant association between IFs and intelligence quotient, CBCL, and NIDA substance involvement (eFigure 1, eFigure 2, and eFigure 3 in Supplement 1).

NKI-RS participants with IFs had higher BMI (mean [SD] BMI: with IF, 27.4 [6.5]; without IF, 24.6 [6.2]; *P* < .001) (eFigure 4A in Supplement 1), which remained significant in adult participants after correcting for age. In a post hoc exploratory analysis, BMI was higher for individuals with T2 hyperintensity and/or cerebral atrophy (mean [SD] BMI, 27.8 [6.3]) vs those without T2 hyperintensity and/or cerebral atrophy mean [SD] BMI, 25.1 [6.4]) (*P* < .001) (eFigure 4B in Supplement 1). This appeared to drive the overall BMI association; rates of other IF types had no significant association with BMI (eFigure 4C in Supplement 1). High BMI (≥25.0) was associated with a somewhat increased risk for IFs for all participants (risk ratio, 1.5; 95% CI, 1.3-1.6); for adults, the risk ration was greater than 1, but the finding was not statistically significant (risk ratio, 1.1; 95% CI, 0.97-1.2). Cumulative probability of common IFs by age in the NKI-RS are in eFigure 5 in Supplement 1.

**Figure 4.**
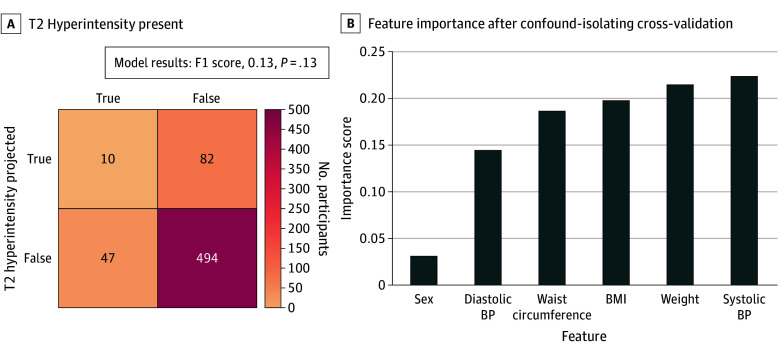
Projection of T2 Hyperintensity From Physiologic Variables in the Nathan Kline Institute–Rockland Sample Using confound-isolating cross-validation to account for the effect of age on the prevalence of incidental findings, a random forest classifier was trained to project the presence or absence of T2 hyperintensity from weight, waist circumference, blood pressure (BP), body mass index (BMI), and birth sex for participants in the Nathan Kline Institute–Rockland Sample.

A projection model was trained using the NKI-RS dataset to estimate the presence of IFs from sex, BMI, systolic and diastolic blood pressure, weight, and waist circumference. After controlling for the dominant age effect using confound-isolating cross-validation,^22^ we found weak evidence of an association (F1 = 0.13; *P* = .13). The features with the best performance for projecting the presence or absence of T2 hyperintensity were weight, systolic blood pressure, body mass index, waist circumference, and diastolic blood pressure, in that order (Figure 4).

### Longitudinal Stability of IFs

Of the NKI-RS participants, 230 pediatric longitudinal participants (107 [47%] female, mean [SD] enrollment age, 11.4 [3.2] years) and 287 adult longitudinal participants (204 [71%] female; mean [SD] enrollment age, 54.2 [10.6] years) were included. Pediatric longitudinal participants returned for 2 follow-up visits, either at 12 and 24 months or at 15 and 30 months, following an initial longitudinal characterization visit (range, 140-1634 days). Adult longitudinal participants returned for 3 follow-up visits at 12, 24, and 36 months following an initial longitudinal characterization visit (range, 154-2642 days). Some longitudinal participants also participated in the core cross-sectional NKI-RS several years prior. In these cases, the core cross-sectional NKI-RS data were also included. MRI retest scans were obtained, ideally, within 3 weeks of characterization visits. Mean radiologist-reported recall—defined as the probability of identifying a baseline reported finding on a longitudinal follow-up scan—was 0.75 within 1 year of baseline and 0.50 at 3 or more years after baseline (eFigure 6 in Supplement 1).

### Motion Artifacts

Radiologist-reported motion artifacts were most prominent in prepubertal children and infrequently reported in adulthood (eFigure 7A in Supplement 1). Rates of radiologist-reported motion decreased from 51 of 155 (32.9%) at age 5 years to 28 of 246 (11.4%) at age 12 years (roughly coinciding with onset of puberty) (eFigure 7A in Supplement 1). At the age of 16 years, motion artifacts were reported among 6 of 159 participants (3.8%), which was consistent with adult rates.

Although participants with radiologist-reported motion had significantly lower Euler numbers in both the NKI-RS (mean Euler number with radiologist-reported motion, −162.3; mean Euler number without, −74.5; *P* < .001) and HBN (mean Euler number with radiologist-reported motion, −562.4; mean Euler number without, −215.7; *P* < .001), the range of Euler numbers was broad in those without radiologist-reported motion (eFigure 7B in Supplement 1). As a result, Euler numbers poorly discriminated scans with clinically relevant motion. Aside from perivascular spaces, which were reported more frequently in NKI-RS adults with radiologist-reported motion than in those without, common category 2 IFs were reported at similar rates in both children and adults regardless of radiologist-reported motion (eTable 8 in Supplement 1).

Motion terciles were defined based on the distribution of Euler numbers at baseline (eg, the high motion tercile was defined as the top third of Euler numbers at baseline). Of the participants in the high motion tercile at baseline, 40 of 72 (55.6%) continued to have motion parameters consistent with the baseline-defined high motion tercile at visit 2. This reduced to 38.1% (16 of 42) at visit 3 and 31.3% (5 of 16) at visit 4 (eFigure 7C in Supplement 1).

### IF Cost of Detection

We used the published 2022 Medicare rate of $368 for acquisition and radiologist review of a noncontrast clinical brain MRI and an estimated $60 fee for radiologist review of scans obtained in a research setting to estimate costs. The approximate cost of detection for category 4 findings was calculated as $87 619/finding and $16 667/finding for clinical and research settings, respectively (eFigure 8 in Supplement 1).

### Interrater Reliability

Of the 517 corated MRI reports, 409 (79.1%) were coded identically between the 2 reviewers. Of the reports that were coded differently between the two reviewers, 72 omitted an IF, 30 had an incorrect IF code, and 2 had differing severity categorizations. The 72 missing IF codes were all category 2 findings, most commonly T2 hyperintensity (11); perivascular spaces (10); and mucosal thickening of the sinuses (8). The eResults in Supplement 1 has further details.

## Discussion

The NKI-RS and HBN are large neuroimaging data-sharing initiatives with robust health phenotyping. Combining these samples yields a highly heterogeneous collective cohort (N = 4072) well-positioned for exploration of IF rates and their health associations. To our knowledge, few past efforts have evaluated the influence of motion on IF detection or attempted to associate Euler numbers with clinically significant motion. Our general findings are in alignment with past efforts: (1) IFs are common, (2) IFs are rarely of urgent concern, and (3) rates of IFs increase with age.

As expected, there were neither differences in rates of IFs for those with mental health diagnoses nor associations with any of the investigated behavioral health or cognitive measures across the lifespan. Common IF findings in children tended to be developmental anatomic variants or extra-axial cysts. Only in instances of mass effect would these findings be expected to directly interrupt brain parenchymal architecture to affect behavior or cognition.

In adults, higher blood pressure, body mass, and related anthropometric indices were associated with the presence of T2 hyperintensities and/or cortical atrophy. T2 hyperintensities and cortical atrophy often co-occur, although it remains unclear whether this results from shared etiologic factors or direct association.^23^ Both increase with age and have been associated with microvascular disease, neuroinflammation, and, in the case of T2 hyperintensities, affective disorders.^24,25,26,27^ Elevated BMI is both an independent and indirect risk factor for cerebrovascular disease.^28^ Our findings may further support that T2 hyperintensities and cortical atrophy warrant consideration in addressing metabolic factors and minimizing cerebrovascular risk factors. However, longer duration longitudinal studies are required to further probe linkages between these IFs and other outcomes, such as stroke.

Given the small group of clinical radiologists involved and their ability to reference past scans and reports, it was surprising that many initially reported IFs were not reported at follow-up. Although we cannot exclude the possibility that radiologists omitted past findings given prior reporting, these results may support less precision in IF reporting. Driving factors may include more subtle aspects of the findings, nonstandard reporting thresholds, or resolution of findings over time.

Although radiologist-reported motion did not statistically impact overall rates of IF reporting, perivascular spaces were more commonly reported in the context of motion. This raises the possibility that motion-related blurring may contribute to false identification of perivascular spaces and may be a consideration with higher motion. However, most radiologist-reported motion was in children who tended to have fewer IFs. Many of the IFs reported in children would have been readily observable in comparison with more subtle IFs acquired in aging.

Our findings also contribute to broader discussion^29,30,31^ regarding MRI screening of neurologically asymptomatic populations. Screening is justified if it is cost-effective and improves patient survival or quality of life.^32^ Approximately 0.2% of children and 1.0% of adults in our merged NKI-RS and HBN data set had IFs that warranted immediate clinical referral. Although outcomes of clinical referral were not obtained in the study, clinical radiologist review of research scans may still be appropriate assuming a standard cost of radiologist review at approximately $50 to $80 per scan. Such a cost is relatively nominal when compared with the cost of obtaining a research scan within the study. From an ethical research standpoint, clinical review of research data with potential clinical implication is also appropriate. However, the potential emotional distress and financial costs incurred in pursuing further clinical work-up should be considered. Conducting broad brain MRI scans in asymptomatic community populations may lead to overidentification of subclinical conditions that would have remained clinically insignificant throughout the remainder of an individual’s life.^33^ Our determined rates of IFs likely do not support MRI screenings in asymptomatic individuals.

Although quantitative classification by Euler number aligned with radiologist-reported motion, the relevance of Euler numbers in clinical interpretability of scans is questionable for 2 primary reasons from our analyses. First, the gold standard radiologist-reported motion had no significant bearing on IF rates, and second, variability in Euler number was significant in scans without radiologist-reported motion.

### Limitations

The present work had several notable limitations. First, neuroradiologists were not instructed to code scans based on set research conventions. We believe this improves clinical generalizability since radiologists may review scans differently if instructed to code data for research purposes. However, the lack of guidelines for radiologists required the coding team to interpret IF types, groupings, clinical severity, and radiologist recommendations—creating potential for error. The establishment of good intercoder reliability minimized these concerns, particularly because areas of rater divergence were often due to omission of category 2 findings embedded in larger radiologist descriptions of the scans rather than the clinical summaries. Approaches to further improve interrater reliability should be considered in future studies.

While we believe our results are generalizable to clinical settings, some limitations remain. A radiologist-reported recommendation of “clinical correlation required” was coded as level 3 since the process of clinical correlation requires some degree of parallel clinical assessment. The same individual may not have required referral in a clinical setting where clinical history and examination could have been performed. Additionally, the HBN and NKI-RS required an hour of scanning, which may have contributed to fatigue and greater motion.

We also cannot rule out that some findings of no clinical significance were unreported. Most IFs were infrequent without power to directly investigate correlations with health phenotyping. Particularly in adults, more severe psychiatric and substance diagnoses were not heavily represented in the NKI-RS. Additionally, we do not have clinical follow-up on participants.

## Conclusions

In this study, IFs were common. While knowing the rates of individual IFs is helpful in establishing normative context, the implications of common IFs remain incompletely understood. Clearly, rates of clinically concerning IFs are low in community settings. Although most IFs have no health associations, white matter hyperintensities and age-related cortical atrophy may raise clinical concern due to their association with metabolic health variables.

## Notes

Supplement 1.
eMethods.

eResults.
**eTable 1.** Full List of Incidental Findings Prior to Merging **eTable 2.** Merged Incidental Findings **eTable 3.** List of Incidental Findings **eTable 4.** Prevalence of Incidental Findings in NKI-RS and HBN Participants **eTable 5.** Risk Ratios for Psychiatric Diagnoses in All NKI-RS Participants **eTable 6.** Risk Ratios for Psychiatric Diagnoses in Adult (Ages ≥22 Years) NKI-RS Participants **eTable 7.** Risk Ratios for Psychiatric Diagnoses in All HBN Participants **eTable 8.** Rates of IF Reporting With and Without Radiologist-Reported Motion **eFigure 1.** IQ Distribution for Individuals With and Without Incidental Findings **eFigure 2.** CBCL Total and Subscale t Score Distributions for Individuals With and Without Incidental Findings **eFigure 3.** NIDA Total Alcohol Substance Involvement Score Distributions for Individuals With and Without Incidental Findings **eFigure 4.** Association of Incidental Findings With Body Mass Index in the NKI-RS **eFigure 5.** Cumulative Probability of Common Incidental Findings by Age in the NKI-RS **eFigure 6.** Longitudinal Stability of Incidental Findings **eFigure 7.** Radiologist-Reported Motion Artifacts and Quantified Motion **eFigure 8.** Estimated Cost of Detecting Category 3 and Category 4 IFs in Clinical and Research Settings
eReferences.

Supplement 2.
Data Sharing Statement
