# Supplementary material for: Age, Motion, Medical, and Psychiatric Associations With Incidental Findings in Brain MRI
Source: JAMA Netw Open. 2024 Feb 13;7(2):e2355901. doi: 10.1001/jamanetworkopen.2023.55901 (PMC10865144; doi:10.1001/jamanetworkopen.2023.55901)
Supplement: Supplement 1. — eMethods. eResults. eTable 1. Full List of Incidental Findings Prior to Merging eTable 2. Merged Incidental Findings eTable 3. List of Incidental Findings eTable 4. Prevalence of Incidental Findings in NKI-RS and HBN Participants eTable 5. Risk Ratios for Psychiatric Diagnoses in All NKI-RS Participants eTable 6. Risk Ratios for Psychiatric Diagnoses in Adult (Ages ≥22 Years) NKI-RS Participants eTable 7. Risk Ratios for Psychiatric Diagnoses in All HBN Participants eTable 8. Rates of IF Reporting With and Without Radiologist-Reported Motion eFigure 1. IQ Distribution for Individuals With and Without Incidental Findings eFigure 2. CBCL Total and Subscale t Score Distributions for Individuals With and Without Incidental Findings eFigure 3. NIDA Total Alcohol Substance Involvement Score Distributions for Individuals With and Without Incidental Findings eFigure 4. Association of Incidental Findings With Body Mass Index in the NKI-RS eFigure 5. Cumulative Probability of Common Incidental Findings by Age in the NKI-RS eFigure 6. Longitudinal Stability of Incidental Findings eFigure 7. Radiologist-Reported Motion Artifacts and Quantified Motion eFigure 8. Estimated Cost of Detecting Category 3 and Category 4 IFs in Clinical and Research Settings eReferences. [file jamanetwopen-e2355901-s001.pdf]

Supplemental Online Content

Tobe RH, Tu L, Roberts M, et al. Age, motion, medical, and psychiatric associations with incidental findings in brain MRI. *JAMA Netw Open.* 2024;7(2):e2355901. doi:10.1001/jamanetworkopen.2023.55901

eMethods.

eResults.

eTable 1. Full List of Incidental Findings Prior to Merging

eTable 2. Merged Incidental Findings

eTable 3. List of Incidental Findings

eTable 4. Prevalence of Incidental Findings in NKI-RS and HBN Participants

eTable 5. Risk Ratios for Psychiatric Diagnoses in All NKI-RS Participants

eTable 6. Risk Ratios for Psychiatric Diagnoses in Adult (Ages ≥22 Years) NKI-RS Participants

eTable 7. Risk Ratios for Psychiatric Diagnoses in All HBN Participants

eTable 8. Rates of IF Reporting With and Without Radiologist-Reported Motion

eFigure 1. IQ Distribution for Individuals With and Without Incidental Findings

eFigure 2. CBCL Total and Subscale t Score Distributions for Individuals With and Without Incidental Findings

eFigure 3. NIDA Total Alcohol Substance Involvement Score Distributions for Individuals With and Without Incidental Findings

eFigure 4. Association of Incidental Findings With Body Mass Index in the NKI-RS

eFigure 5. Cumulative Probability of Common Incidental Findings by Age in the NKI-RS

eFigure 6. Longitudinal Stability of Incidental Findings

eFigure 7. Radiologist-Reported Motion Artifacts and Quantified Motion

eFigure 8. Estimated Cost of Detecting Category 3 and Category 4 IFs in Clinical and Research Settings

eReferences.

This supplemental material has been provided by the authors to give readers additional information about their work.

© 2024 Tobe RH et al. *JAMA Network Open.*

eMethods

Measures

The Kiddie-Schedule for Affective Disorders and Schizophrenia (KSADS) for School-Aged Children Present and Lifetime Version (PL)[1] is a semi-structured research diagnostic assessment designed to assess current and past episodes of psychopathology in children and adolescents, according to DSM-IV criteria.

The Structured Clinical Interview for DSM-IV Disorders (SCID-IV)[2] is a semi-structured psychiatric diagnostic interview designed to assess current and past episodes of psychopathology in adults, according to DSM-IV criteria.

The Child Behavior Checklist (CBCL)[3] is used to detect behavioral and emotional problems in children and adolescents. The CBCL is completed by parents or surrogates and consists of 113 questions scored on a three-point Likert scale (0 = absent, 1 = occurs sometimes, 2=occurs often).

The Adult Self Report (ASR)[4] is used to assess adaptive functioning, problems, and substance use in adults. The ASR consists of 126 questions scored on a three-point Likert scale (0 = not true, 1 = somewhat or sometimes true, 2 = very true or often true).

The Wechsler Abbreviated Scale of Intelligence—Second Edition (WASI-II)[5] is an individually-administered general intelligence test designed to assess overall cognitive capabilities. It includes four subtests (Block Design [13 items], Vocabulary [31 items], Matrix Reasoning [30 items], and Similarities [24 items]) and yields a full scale intelligence quotient (FSIQ).

The Quick Screen was adapted by NIDA from a single-question screen for drug use in primary care[6]. The NIDA-modified Alcohol, Smoking, and Substance Involvement Screening Test (ASSIST)[7] was used in conjunction with the Quick Screen. The NIDA Quick Screen/ASSIST is an 8-item self-report screen assessing frequency, urge, as well as health, functional and economic consequences of use.

Magnetic Resonance Imaging and Reporting

All scanning took place on Siemens 3.0T scanners with 32-head channel coils. NKI-RS participants were scanned on a Siemens Tim Trio at the Nathan S. Kline Institute for Psychiatric Research (NKI). HBN participants were scanned on a Siemens Tim Trio at the Rutgers University Brain Imaging Center (RUBIC), a Prisma Fit at the Weill Cornell Medicine Citigroup Biomedical Imaging Center (CBIC), or a Prisma scanner at the City University of New York (CUNY) Advanced Science Research Center (ASRC)'s Magnetic Resonance Imaging Facility (MRIF).

The T2-FLAIR for radiological reading had a slice thickness of 3mm and was in plane resolution of $1mm^2$.

The clinical neuroradiology core was composed of three primary clinical neuroradiologists who reviewed greater than 95% of structural scans throughout the duration of the studies. Each neuroradiologist reviewed similar proportions of scans for each study rather than a particular neuroradiologist having primary assignment to a specific initiative. Though all neuroradiologists reviewed a significant proportion of the scans (i.e., at least 20% for each study) between protocols, there was no controlling for the total number of scans reviewed by each neuroradiologist. For longitudinal participants, the cases were not necessarily assigned to the neuroradiologist who reviewed the prior scan(s).

© 2024 Tobe RH et al. *JAMA Network Open.*

eResults

Though inter-rater reliability was considered good, particularly in the context of high IF rates in the NKI-RS and multiple participants having multiple IFs in a single report, the results were lower than expected. On qualitative debriefing with the raters in evaluating discrepancies, two primary vulnerabilities for disparate coding were identified. First, neuroradiologist reports consisted of a larger descriptive synopsis of general findings followed by a brief clinical summary of those findings with recommendations. Accordingly, a neuroradiologist may list a series of clinically insignificant IF(s) in the much larger descriptive synopsis but not in the summary. These seemed harder for raters to consistently identify and is the likely reason all missing IF codes were category 2 (rather than category 3 or 4) severity findings. Second, several neuroradiologist-reported IFs were composed of two coded items (e.g., age-related cerebral atrophy). In this context, raters could differ in their coding of age-related changes, cerebral atrophy, or both. The raters had excellent agreement on severity categorization (with only 2 IFs differing among the 517 reports). This was likely due to the neuroradiology groups producing precise clinical synopses with recommendations, as needed.

© 2024 Tobe RH et al. *JAMA Network Open.*

eTable 1. Full List of Incidental Findings Prior to Merging

Incidental finding
6-7 mm nonspecific focus of T2 hyperintensity within the basal ganglia
Abnormal shape of the orbital globes
Abnormal signal in the dentate nuclei of the cerebellum bilaterally
Age related changes
Agenesis of the corpus callosum
Air-fluid level in the sinus
Anterolisthesis
AP elongation of the orbital globe
Arachnoid cyst
Arachnoid/intraventricular cyst with mass effect
Areas of demyelination that may be post ischemic or post inflammatory in nature
Arrested pneumatization in the right mastoid tip
Arteriovenous malformation
Asymmetric lateral ventricles
Asymmetric pneumatization of the petrous apices
Asymmetric temporal horns
Basal ganglia lesion
Basal ganglia mineral depositions
Bilateral cervical adenopathy
Bilateral ethmoid sinusitis
Bilateral maxillary sinusitis
Brachycephaly
Brainstem lesion
Calcifications
Calvarial defect in the left frontal bone parasagittal high convexity with an overlying nodule
Calvarial hyperostosis
Cavernous angioma vs. dystrophic calcification of other etiologies
Cavernous malformation
Cavum septum pellucidum, Cavum vergae
Cavum velum interpositum cysts
Cerebellar anomaly
Cerebellar atrophy
Cerebellar hypoplasia, volume loss, signal abnormality
Cerebellar lesion
Cerebellar tonsillar ectopia
Cerebral cystic lesion
Cerebral lesion
Cerebral lipoma
Cerebral mass
Cervical cord compression, cervical cord indentation
Cervical disc bulging/herniation
Chiari I malformation
Choroid plexus cysts
Choroidal fissure cysts
Chronic microvascular changes
Chronic microvascular ischemic disease, small vessel ischemic disease
Chronic mucosal changes in the maxillary sinuses
Chronic white matter changes in periventricular regions
Concha bullosa
Cortical atrophy
CSF Pulsations
Cystic changes in the parotid glands
Degenerative changes in the visualized cervical spine

© 2024 Tobe RH et al. *JAMA Network Open.*

Developmental venous anomaly
Developmental venous anomaly versus perivascular space in the right frontal lobe
Developmental venous anomaly vs. perivascular space
Deviation of nasal septum
Dilated central canal
Dilated cortical medullary vein
Dilation of bilateral lateral ventricles
Disc herniation/bulging is seen at C3-4 and C4-5 slightly indenting the spinal cord
Edema in right hippocampus
Empty sella
Encephalocele
Encephalomalacia
Endplate osteophytes
Enlarged cortical sulci
Enlarged sella (turcica)
Enlarged tonsils
Enlarged ventricular system
Extra-axial collection
Extra-axial mass seen in the left parasagittal high convexity
Eye globe abnormalities
Findings suggestive of intracranial hypertension
Flair hyperintensity
Flow void
Fluid intensity present in the sinus
Fluid or granulation tissue in the petrous apex
Fluid/granulation tissue in mastoid air cells
Focal cortical dysplasia
Foci of flair hyperintensity throughout the subcortical white matter
Focus of high signal along anterior margin
Frontal lobe lesion
Gliosis
Granulation tissue in the mastoid air cells from prior inflammation
Gray matter heterotopia
Head and neck findings
Heterogeneous signal lesion
High T1 signal collection seen within the right sphenoid sinus that follows the signal of fat
Hydrocephalus
Hypertrophy of the posterior longitudinal ligament
Hypogenesis of the corpus callosum
Increased CSF space
Increased signal of the nasal turbinates
Infarction
Inferior displacement of orbital floor
Internal auditory canal lesion
Involutional changes
Large pineal cyst with mass effect
Low craniofacial ratio
Malformations
Mastoid effusions
Mastoid lesion
Mastoiditis
Mega cisterna magna
Meningioma
Minimal nonspecific white matter changes right frontal region
Morphologic abnormality of cervical spine
Mucosal opacification

Mucosal thickening, mucoperiosteal thickening
Mucous retention cyst
Narrowing of the foramen magnum
Nasal turbinates tissue prominence
Neoplastic process vs. cortical dysplasia
Neuroepithelial cyst
Neuroglial cyst
Nondominant left jugular system
Nonspecific foci of bright signal on flair images
Nonspecific white matter lesions concerning for sequela of infection, inflammation, ischemia, dysmyelination, or demyelination
Occipital lesion
Occipital mass
Old cortical infarction
Old lacunar infarction
Parafalcine calcifications
Paranasal sinus disease
Parotid cysts
Parotid mass
Pars intermedia cyst, Rathke cleft cyst
Partial opacification of the sinuses
Partially empty sella configuration
Perivascular spaces, Virchow-Robin spaces
Periventricular cystic lesion
Periventricular lesion
Periventricular nodular heterotopia
Petrous apex effusion
Pineal cyst
Pineal lesion
Pituitary abnormality
Pituitary cyst
Pituitary lesion
Polymicrogyria
Porencephalic cyst
Possible glial neoplasm
Posterior fossa arachnoid cyst vs. mega cisterna magna
Prominent adenoids
Prominent cervical lymph nodes
Prominent CSF space
Prominent lateral ventricles
Prominent pituitary gland
Prominent retrocerebellar/retrovermian cistern
Prominent retrocerebellar/retrovermian cistern vs. arachnoid cyst without significant mass effect
Prominent tonsils
Prominent Virchow-Robin space
Remote ischemic vs. infectious or inflammatory/ demyelinating etiology
Retrosplenial cavum velum interpositum
Reversal of cervical lordosis
Reversal of curvature in the cervical spine
Reverse cervical lordosis
Scalp Lipoma
Scattered periventricular and subcortical focal signal alterations
Sebaceous cysts
Sellar/suprasellar cystic lesion
Septal deviation
Single punctate high FLAIR signal

© 2024 Tobe RH et al. *JAMA Network Open.*

Sinus cystic lesion
Sinusitis
Small vessel changes of aging noted in the cerebral white matter
Soft Tissue Mass
Sphenoid sinusitis
Spinal stenosis, disc space narrowing
Spondylotic changes, spondylosis
Subependymal cyst
Superior funneling of the aqueduct
Susceptibility artifact concerning for metal in scalp/face
Suspected cavernous malformation
Syringomyelia
Syrinx
T1 hyperintensity
T1 hyperintensity of the choroid plexus in the lateral ventricles
T1 hypointensity
T1/T2 hyperintense small cyst corresponding to the right fossa of Rosenmuller
T2 hyperintensity
Thecal sac indentation
Thickened nerve complexes in the cerebellopontine angle
Thornwaldt cyst
Unilateral dilation of lateral ventricle
Uvula enlargement
Vascular abnormalities
Venous angioma
Ventricular asymmetry
Ventriculomegaly
Vertebrobasilar dolichoectasia
Very small focus of low signal in the right lobe of the pituitary gland
White matter abnormalities concerning for infection, inflammation, or ischemic injury
White matter changes of aging

© 2024 Tobe RH et al. *JAMA Network Open.*

eTable 2. Merged Incidental Findings

Incidental finding
**Age related changes**
Age related changes
Small vessel changes of aging noted in the cerebral white matter
White matter changes of aging
**Arachnoid cyst**
Arachnoid cyst
Arachnoid/intraventricular cyst with mass effect
**Asymmetric lateral ventricles**
Asymmetric lateral ventricles
Asymmetric temporal horns
Unilateral dilation of lateral ventricle
Ventricular asymmetry
**Basal ganglia lesion**
Basal ganglia lesion
Basal ganglia mineral depositions
**Cerebellar anomaly**
Abnormal signal in the dentate nuclei of the cerebellum bilaterally
Cerebellar anomaly
Cerebellar lesion
**Cerebellar atrophy**
Cerebellar atrophy
Cerebellar hypoplasia, volume loss, signal abnormality
**Cerebral atrophy**
Cerebral atrophy
Cortical atrophy
Enlarged cortical sulci
Involutional changes
**Cerebral lesion**
Cerebral lesion
Cerebral lipoma
Frontal lobe lesion
Neuroglial cyst
Occipital lesion
**Choroid plexus cysts**
Choroid plexus cysts
Choroidal fissure cysts
**Chronic microvascular ischemic disease, small vessel ischemic disease**
Chronic microvascular changes
Chronic microvascular ischemic disease, small vessel ischemic disease
**Chronic white matter changes in periventricular regions**
Chronic white matter changes in periventricular regions
Periventricular lesion
Scattered periventricular and subcortical focal signal alterations
**Degenerative changes in the visualized cervical spine**
Anterolisthesis
Cervical cord compression, cervical cord indentation
Cervical disc bulging/herniation
Degenerative changes in the visualized cervical spine
Disc herniation/bulging is seen at C3-4 and C4-5 slightly indenting the spinal cord
Endplate osteophytes
Hypertrophy of the posterior longitudinal ligament
Reversal of cervical lordosis
Reversal of curvature in the cervical spine

© 2024 Tobe RH et al. *JAMA Network Open.*

Reverse cervical lordosis
Spinal stenosis, disc space narrowing
Spondylotic changes, spondylosis
Thecal sac indentation

Deviation of nasal septum

Deviation of nasal septum
Septal deviation

Empty sella

Empty sella
Enlarged sella (turcica)
Partially empty sella configuration

Eye globe abnormalities

Abnormal shape of the orbital globes
AP elongation of the orbital globe
Eye globe abnormalities

Focal cortical dysplasia

Focal cortical dysplasia
Gray matter heterotopia
Periventricular nodular heterotopia

Granulation tissue in the mastoid air cells from prior inflammation

Fluid/granulation tissue in mastoid air cells
Granulation tissue in the mastoid air cells from prior inflammation

Head and neck findings

Head and neck findings
Parotid cysts
Parotid mass
Scalp lipoma
Sebaceous cysts
Soft tissue mass

Hypogenesis of the corpus callosum

Agenesis of the corpus callosum
Hypogenesis of the corpus callosum

Infarction

Infarction
Old cortical infarction
Old lacunar infarction

Mastoiditis

Mastoid effusions
Mastoid lesion
Mastoiditis

Mega cisterna magna

Mega cisterna magna
Posterior fossa arachnoid cyst vs. mega cisterna magna
Prominent retrocerebellar/retrovermian cistern
Prominent retrocerebellar/retrovermian cistern vs. arachnoid cyst without significant mass effect

Meningioma

Extra-axial mass seen in the left parasagittal high convexity
Meningioma

Mucosal opacification

Air-fluid level in the sinus
Fluid intensity present in the sinus
Mucosal opacification
Partial opacification of the sinuses

Mucosal thickening, mucoperiosteal thickening

Chronic mucosal changes in the maxillary sinuses
Increased signal of the nasal turbinates

© 2024 Tobe RH et al. *JAMA Network Open.*

Mucosal thickening, mucoperiosteal thickening
Nasal turbinates tissue prominence
Perivascular spaces, Virchow-Robin spaces
Developmental venous anomaly vs. perivascular space
Developmental venous anomaly vs. perivascular space in the right frontal lobe
Perivascular spaces, Virchow-Robin spaces
Prominent Virchow-Robin space
Petrous apex effusion
Fluid or granulation tissue in the petrous apex
Petrous apex effusion
Pineal cyst
Large pineal cyst with mass effect
Pineal cyst
Pituitary abnormality
Pituitary abnormality
Pituitary lesion
Prominent pituitary gland
Very small focus of low signal in the right lobe of the pituitary gland
Pituitary cyst
Pars intermedia cyst, Rathke cleft cyst
Pituitary cyst
Sellar/suprasellar cystic lesion
Possible glial neoplasm
Cerebral cystic lesion
Cerebral mass
Neoplastic process vs. cortical dysplasia
Neuroepithelial cyst
Occipital mass
Periventricular cystic lesion
Possible glial neoplasm
Prominent cervical lymph nodes
Bilateral cervical adenopathy
Prominent cervical lymph nodes
Prominent CSF space
Increased CSF space
Prominent CSF space
Prominent tonsils
Enlarged tonsils
Prominent tonsils
Sinus cystic lesion
Mucous retention cyst
Sinus cystic lesion
Thornwaldt cyst
Sinusitis
Bilateral ethmoid sinusitis
Bilateral maxillary sinusitis
Concha bullosa
Paranasal sinus disease
Sinusitis
Sphenoid sinusitis
Syringomyelia
Syringomyelia
Syrinx
T2 hyperintensity
6-7 mm nonspecific focus of T2 hyperintensity within the basal ganglia
Flair hyperintensity

© 2024 Tobe RH et al. *JAMA Network Open.*

Foci of flair hyperintensity throughout the subcortical white matter
Minimal nonspecific white matter changes right frontal region
Nonspecific foci of bright signal on flair images
Single punctate high FLAIR signal
T2 hyperintensity
**Vascular abnormalities**
Arteriovenous malformation
Calcifications
Cavernous angioma vs. dystrophic calcification of other etiologies
Cavernous malformation
Developmental venous anomaly
Dilated cortical medullary vein
Flow void
Nondominant left jugular system
Suspected cavernous malformation
Vascular abnormalities
Venous angioma
Vertebrobasilar dolichoectasia
**Ventriculomegaly**
Dilation of bilateral lateral ventricles
Enlarged ventricular system
Hydrocephalus
Prominent lateral ventricles
Unilateral dilation of lateral ventricle
Ventriculomegaly
**White matter abnormalities concerning for infection, inflammation, or ischemic injury**
Nonspecific white matter lesions concerning for sequela of infection, inflammation, ischemia, dysmyelination, or demyelination
White matter abnormalities concerning for infection, inflammation, or ischemic injury

Similar IF terms were merged. Gray subheadings indicate the IF term that was retained. If an IF term was not merged with any other IF terms, it is not included in eTable 2.

© 2024 Tobe RH et al. *JAMA Network Open.*

eTable 3. List of Incidental Findings

Incidental finding
**Brain-based**
Age related changes
Arachnoid cyst
Asymmetric lateral ventricles
Basal ganglia lesion
Brainstem lesion
Cavum septum pellucidum, Cavum vergae
Cavum velum interpositum cysts
Cerebellar anomaly
Cerebellar atrophy
Cerebellar tonsillar ectopia
Cerebral atrophy
Cerebral lesion
Chiari I malformation
Choroid plexus cysts
Chronic microvascular ischemic disease, small vessel ischemic disease
Chronic white matter changes in periventricular regions
Empty sella
Encephalomalacia
Focal cortical dysplasia
Gliosis
Heterogeneous signal lesion
Hypogenesis of the corpus callosum
Infarction
Mega cisterna magna
Meningioma
Parafalcine calcifications
Perivascular spaces, Virchow-Robin spaces
Pineal cyst
Pineal lesion
Pituitary abnormality
Pituitary cyst
Possible glial neoplasm
Prominent CSF space
Retrosplenial cavum velum interpositum
Subependymal cyst
T1 hyperintensity
T1 hyperintensity of the choroid plexus in the lateral ventricles
T1 hypointensity
T2 hyperintensity
Thickened nerve complexes in the cerebellopontine angle
Vascular abnormalities
Ventriculomegaly
White matter abnormalities concerning for infection, inflammation, or ischemic injury
**Not brain-based**
Arrested pneumatization in the right mastoid tip
Asymmetric pneumatization of the petrous apices
Brachycephaly
Calvarial defect in the left frontal bone parasagittal high convexity with an overlying nodule
Calvarial hyperostosis
Degenerative changes in the visualized cervical spine
Deviation of nasal septum
Eye globe abnormalities

© 2024 Tobe RH et al. *JAMA Network Open.*

Granulation tissue in the mastoid air cells from prior inflammation
Head and neck findings
High T1 signal collection seen within the right sphenoid sinus that follows the signal of fat
Inferior displacement of orbital floor
Internal auditory canal lesion
Low craniofacial ratio
Mastoiditis
Mucosal opacification
Mucosal thickening, mucoperiosteal thickening
Narrowing of the foramen magnum
Petrous apex effusion
Prominent adenoids
Prominent cervical lymph nodes
Prominent tonsils
Sinus cystic lesion
Sinusitis
Syringomyelia
Uvula enlargement

© 2024 Tobe RH et al. *JAMA Network Open.*

eTable 4. Prevalence of Incidental Findings in NKI-RS and HBN Participants

Incidental finding	Total participant no. (%)	Category 2 no. of instances (%)	Category 3 no. of instances (%)	Category 4 no. of instances (%)
**NKI-RS**				
T2 hyperintensity	424 (32.6)	400 (89.3)	46 (10.3)	2 (0.4)
Perivascular or VR spaces	165 (12.7)	165 (100.0)	0 (0.0)	0 (0.0)
Empty sella	156 (12.0)	156 (100.0)	0 (0.0)	0 (0.0)
Chronic microvascular or small ischemic disease	153 (11.8)	152 (98.7)	2 (1.3)	0 (0.0)
Pineal cyst	51 (3.9)	49 (94.2)	3 (5.8)	0 (0.0)
Cerebral atrophy	42 (3.2)	42 (100.0)	0 (0.0)	0 (0.0)
Cerebellar tonsillar ectopia	32 (2.5)	32 (100.0)	0 (0.0)	0 (0.0)
Age related changes	15 (1.2)	15 (100.0)	0 (0.0)	0 (0.0)
Cerebral lesion	15 (1.2)	13 (81.3)	3 (18.8)	0 (0.0)
Pituitary abnormality	14 (1.1)	5 (35.7)	9 (64.3)	0 (0.0)
Vascular Abnormalities	14 (1.1)	12 (80.0)	3 (20.0)	0 (0.0)
Mega cisterna magna	13 (1.0)	13 (100.0)	0 (0.0)	0 (0.0)
Choroid plexus cysts	12 (0.92)	12 (100.0)	0 (0.0)	0 (0.0)
Chiari I malformation	9 (0.69)	5 (55.6)	4 (44.4)	0 (0.0)
Prominent CSF space	9 (0.69)	9 (100.0)	0 (0.0)	0 (0.0)
Cavum septum pellucidum, Cavum vergae	8 (0.62)	8 (100.0)	0 (0.0)	0 (0.0)
Infarction	7 (0.54)	6 (85.7)	1 (14.3)	0 (0.0)
Arachnoid cyst	7 (0.54)	7 (87.5)	1 (12.5)	0 (0.0)
Pituitary cyst	7 (0.54)	4 (44.4)	5 (55.6)	0 (0.0)
White matter changes in periventricular regions	7 (0.54)	7 (100.0)	0 (0.0)	0 (0.0)
Encephalomalacia	7 (0.54)	6 (66.7)	3 (33.3)	0 (0.0)
Asymmetric lateral ventricles	7 (0.54)	7 (100.0)	0 (0.0)	0 (0.0)
Ventriculomegaly	6 (0.46)	6 (100.0)	0 (0.0)	0 (0.0)
Possible glial neoplasm	5 (0.38)	0 (0.0)	1 (20.0)	4 (80.0)
**HBN**				
Pineal cyst	49 (1.8)	44 (89.8)	4 (8.2)	1 (2.0)
Empty sella	44 (1.6)	43 (97.7)	0 (0.0)	1 (2.3)
Mega cisterna magna	24 (0.9)	21 (87.5)	3 (12.5)	0 (0.0)
Arachnoid cyst	17 (0.6)	13 (76.5)	3 (17.6)	1 (5.9)
Choroid plexus cysts	15 (0.5)	14 (93.3)	1 (6.7)	0 (0.0)
Chiari I malformation	10 (0.4)	8 (80.0)	2 (20.0)	0 (0.0)
Pituitary abnormality	9 (0.3)	1 (11.1)	8 (88.9)	0 (0.0)
Perivascular spaces, Virchow-Robin spaces	7 (0.3)	7 (100.0)	0 (0.0)	0 (0.0)
Asymmetric lateral ventricles	6 (0.2)	5 (83.3)	1 (16.7)	0 (0.0)
T2 hyperintensity	6 (0.2)	6 (100.0)	0 (0.0)	0 (0.0)
Prominent CSF space	6 (0.2)	6 (100.0)	0 (0.0)	0 (0.0)
Possible glial neoplasm	5 (0.2)	1 (20.0)	2 (40.0)	2 (40.0)
Ventriculomegaly	6 (0.2)	4 (66.7)	1 (16.7)	1 (16.7)
Cavum septum pellucidum, Cavum vergae	5 (0.2)	5 (100.0)	0 (0.0)	0 (0.0)

© 2024 Tobe RH et al. *JAMA Network Open.*

eTable 5. Risk ratios for psychiatric diagnoses in all NKI-RS participants

Psychiatric Diagnosis	Risk Ratio
Alcohol Abuse	1.27
Anxiety Disorder NOS	0.91
Attention-Deficit/Hyperactivity Disorder	0.58
Depressive Disorder NOS	1.21
Drug Abuse	1.09
Eating Disorder	1.19
Enuresis	0.46
Generalized Anxiety Disorder	0.98
Major Depressive Disorder	1.17
Obsessive-Compulsive Disorder	0.66
Panic Disorder	1.14
Posttraumatic Stress Disorder	1.29
Specific Phobia	0.97
Tic Disorder	0.44

© 2024 Tobe RH et al. *JAMA Network Open.*

eTable 6. Risk ratios for psychiatric diagnoses in adult (ages 22+) NKI-RS participants

Psychiatric diagnosis	Risk ratio
Alcohol Abuse	1.03
Anxiety Disorder NOS	0.88
Attention-Deficit/Hyperactivity Disorder	0.74
Depressive Disorder NOS	1.17
Drug Abuse	0.95
Eating Disorder	0.98
Generalized Anxiety Disorder	1.08
Major Depressive Disorder	1.01
Obsessive-Compulsive Disorder	0.59
Panic Disorder	1.08
Posttraumatic Stress Disorder	1.13
Specific Phobia	1.06

© 2024 Tobe RH et al. *JAMA Network Open.*

eTable 7. Risk ratios for psychiatric diagnoses in all HBN participants

Psychiatric disorder	Risk ratio
Adjustment Disorders	0.94
Anxiety Disorder NOS	0.80
Attention-Deficit/Hyperactivity Disorder	0.95
Autism Spectrum Disorder	1.24
Elimination Disorder	0.96
Generalized Anxiety Disorder	0.96
Intellectual Disability	0.46
Language Disorder	1.13
Learning Disorder	1.13
Major Depressive Disorder	0.71
Obsessive-Compulsive Disorder	1.44
Oppositional Defiant Disorder	0.72
Persistent Depressive Disorder (Dysthymia)	0.60
Separation Anxiety	1.12
Social Anxiety (Social Phobia)	0.64
Specific Phobia	1.06
Speech Sound Disorder	0.41
Tic Disorder	0.95

© 2024 Tobe RH et al. *JAMA Network Open.*

eTable 8. Rates of IF Reporting With and Without Radiologist-Reported Motion

Incidental finding	Incidental finding rate		
	With radiologist-reported motion artifacts (%)	Without radiologist-reported motion artifacts (%)	*p* value
**NKI-RS adults (ages 22+)**			
Cerebellar tonsillar ectopia	7.8	0.8	.17
Cerebral atrophy	5.9	3.5	> .99
Chronic microvascular or small ischemic disease	11.8	18.5	> .99
Empty sella	3.9	13.0	.15
Perivascular or VR spaces	37.3	9.3	< .001
Pineal cyst	2.0	3.6	> .99
T2 hyperintensity	43.1	46.6	> .99
**NKI-RS children (ages 6-21)**			
Cerebellar tonsillar ectopia	5.4	1.4	.19
Cerebral atrophy	0.0	0.0	> .99
Chronic microvascular or small ischemic disease	0.0	0.1	> .99
Empty sella	1.3	3.8	.84
Perivascular or VR spaces	4.0	1.9	.84
Pineal cyst	3.4	2.7	> .99
T2 hyperintensity	6.0	5.6	> .99
**HBN children (ages 6-21)**			
Arachnoid cyst	0.2	0.7	> .99
Chiari I malformation	0.2	0.4	> .99
Choroid plexus cysts	0.6	0.5	> .99
Empty sella	0.8	1.8	.58
Mega cisterna magna	1.6	0.7	.58
Pineal cyst	1.2	1.9	> .99

Rates of commonly reported IFs are represented with and without radiologist-reported motion in NKI-RS adults, NKI-RS children, and HBN children. The only IF which was reported more frequently in the context of radiologist-reported motion artifacts was perivascular (Virchow-Robin) spaces. No IFs were less commonly reported in the context of radiologist-reported motion.

© 2024 Tobe RH et al. *JAMA Network Open.*

eFigure 1. IQ Distribution for Individuals With and Without Incidental Findings

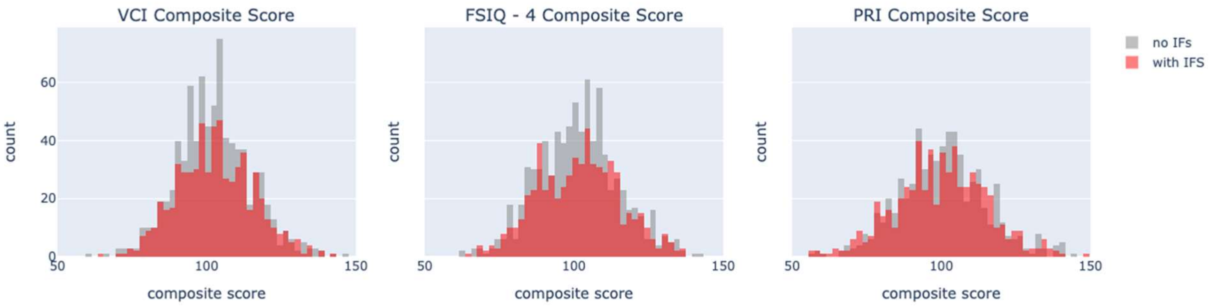

© 2024 Tobe RH et al. *JAMA Network Open.*

eFigure 2. CBCL Total and Subscale *t*-score Distributions for Individuals With and Without Incidental Findings

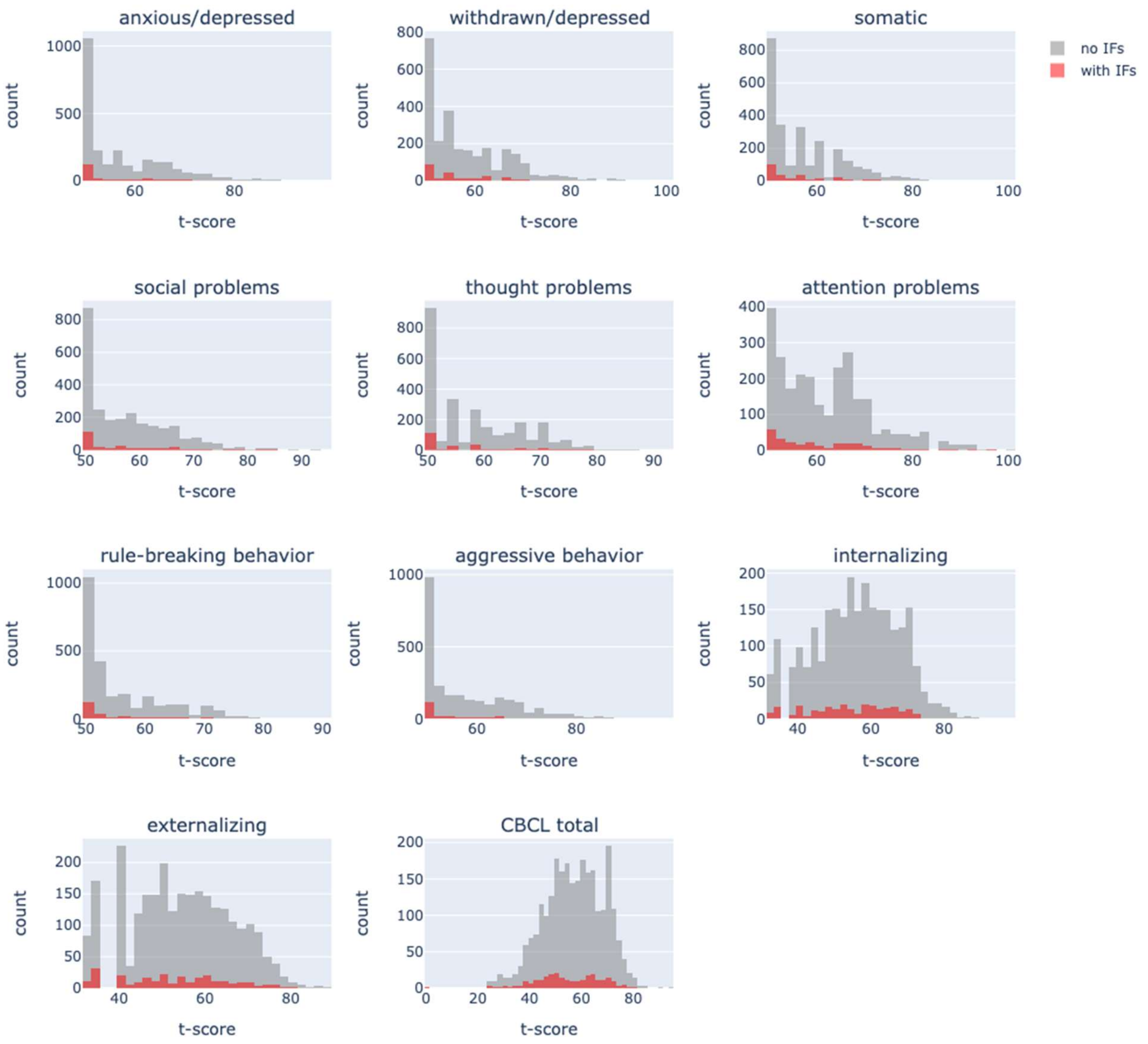

© 2024 Tobe RH et al. *JAMA Network Open.*

eFigure 3. NIDA Total Alcohol Substance Involvement Score Distributions for Individuals With and Without Incidental Findings

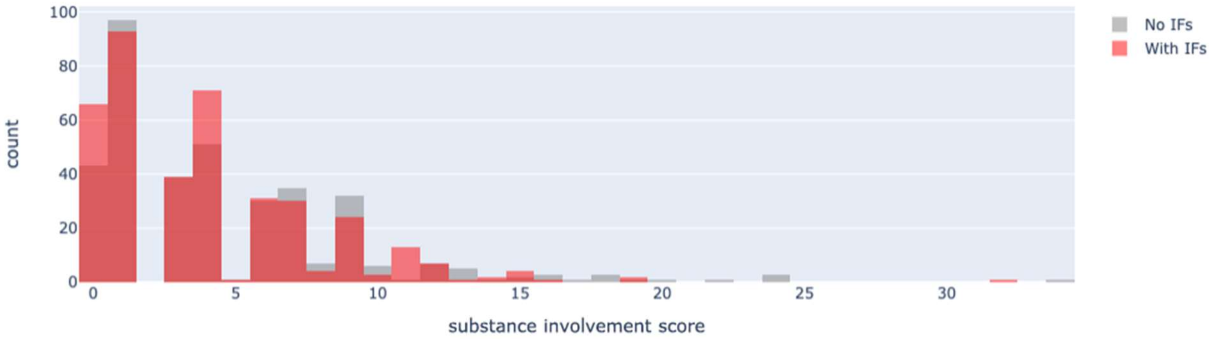

© 2024 Tobe RH et al. *JAMA Network Open.*

eFigure 4. Relationship of Incidental Findings and Body Mass Index in the NKI-RS

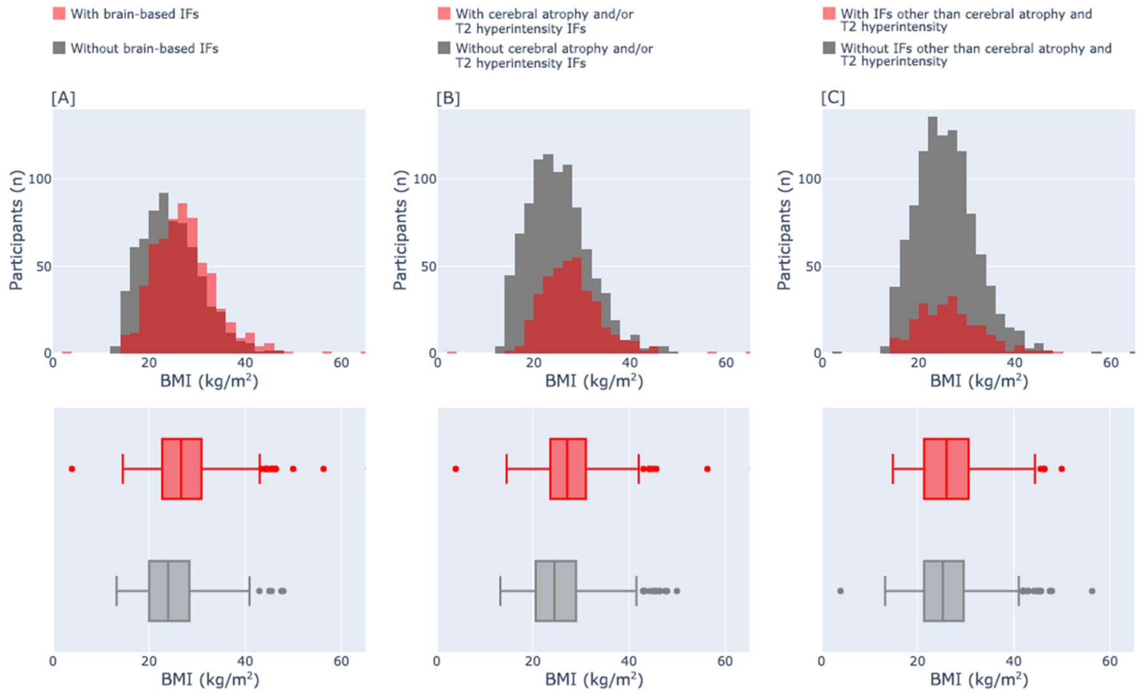

The number of participants (*n*) are represented by their BMI (kg/m^2) with and without [A] any brain-based IFs, [B] IFs of cerebral atrophy and/or T2 hyperintensity, and [C] IFs other than cerebral atrophy and T2 hyperintensity.

© 2024 Tobe RH et al. *JAMA Network Open.*

eFigure 5. Cumulative Probability of Common Incidental Findings by Age in the NKI-RS

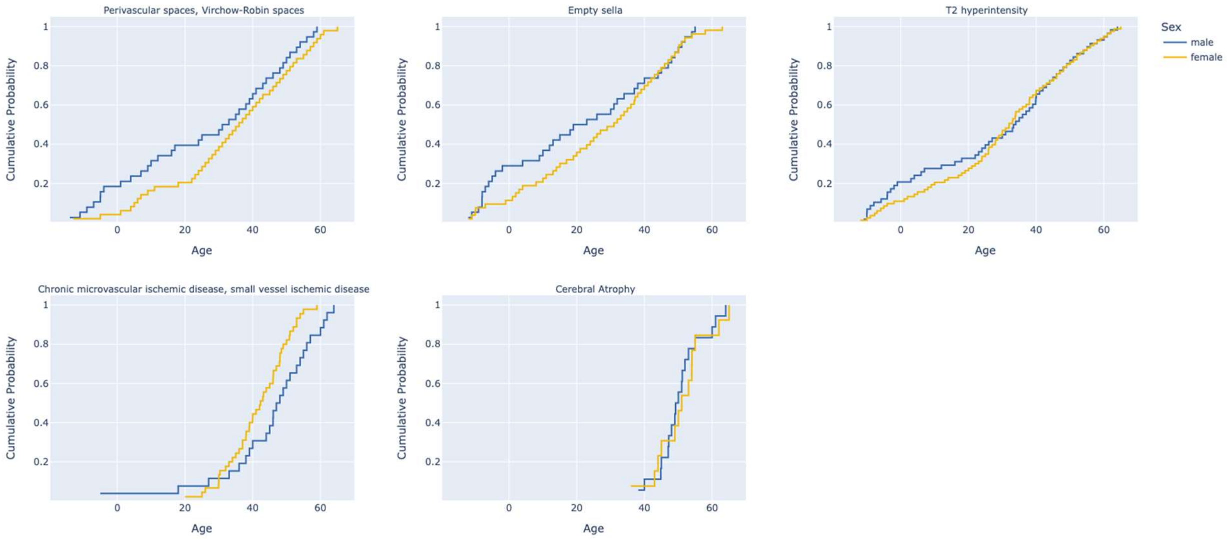

© 2024 Tobe RH et al. *JAMA Network Open.*

eFigure 6. Longitudinal Stability of Incidental Findings

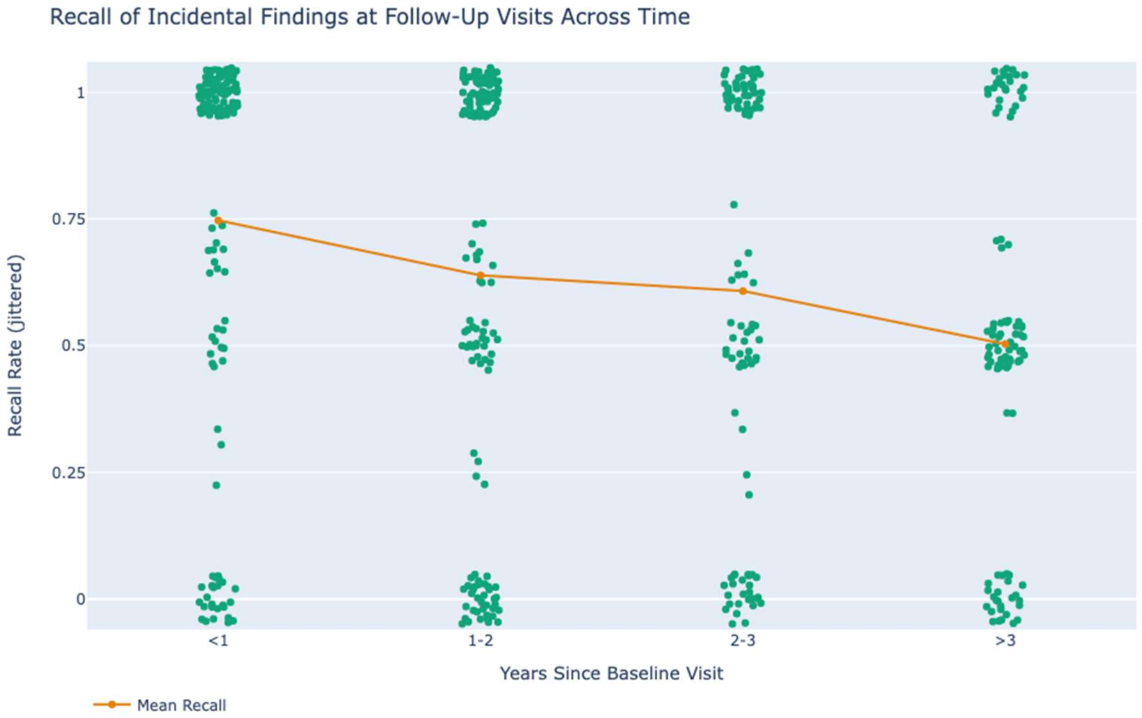

The recall rate, or fraction of findings recorded at baseline that were also recorded at follow-up, is represented over time. The mean recall rate for each time period is annotated on the plots as an orange marker. The mean recall rate decreased over time and ranged from 0.75 within 1 year of baseline and 0.50 at 3 or more years after baseline.

eFigure 7. Radiologist-Reported Motion Artifacts and Quantified Motion

[A] Number of Participants With and Without Radiologist-Reported Motion Artifacts

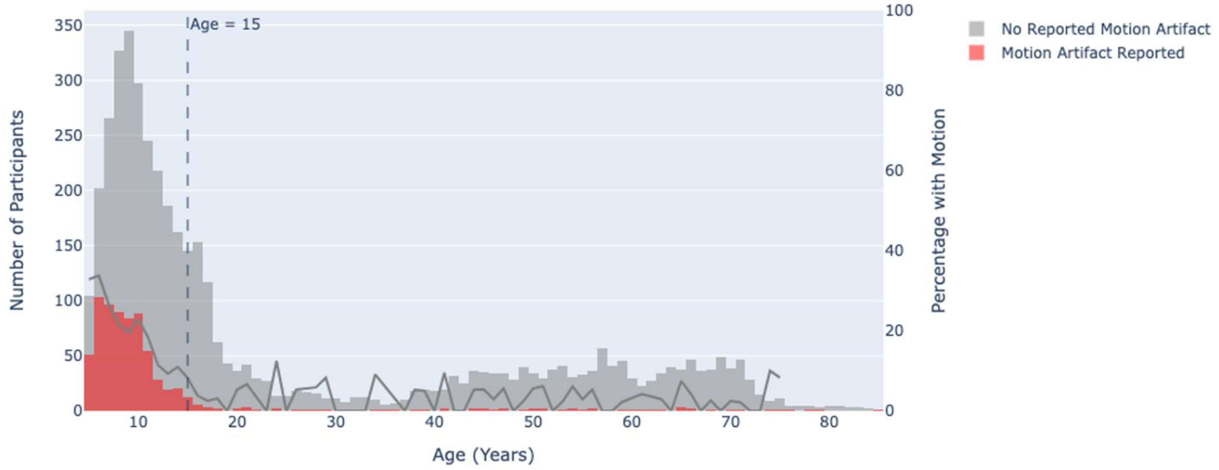

[B] Euler Numbers by Population Group and Motion

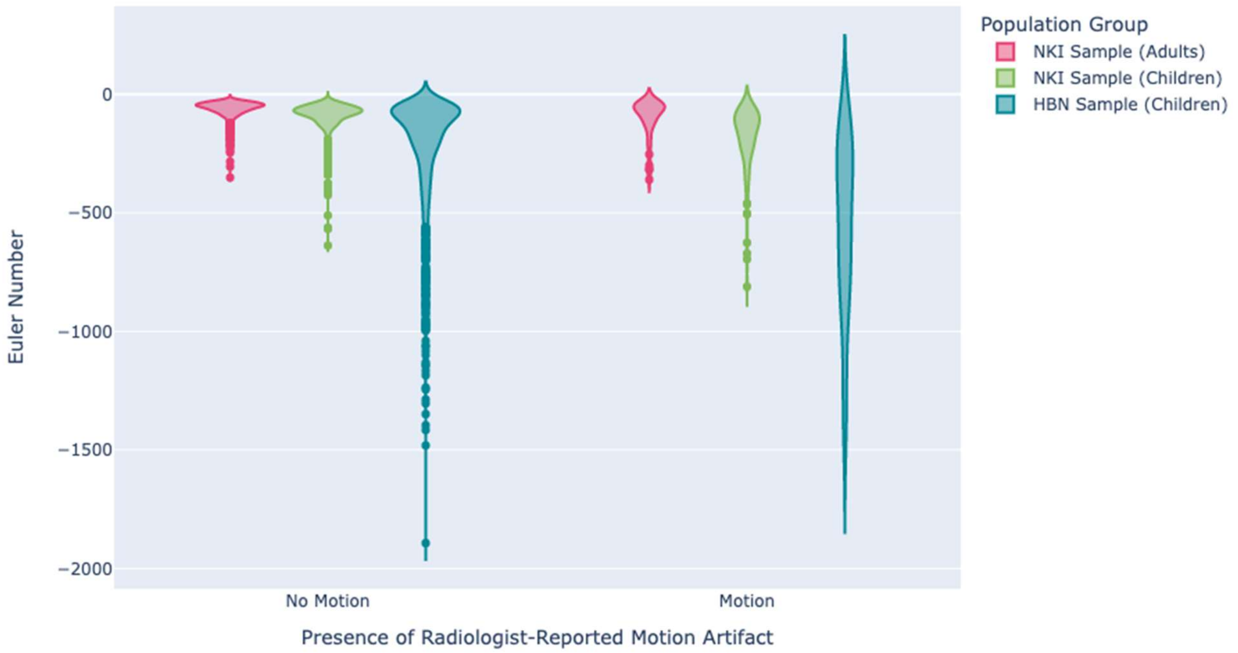

© 2024 Tobe RH et al. *JAMA Network Open.*

[C] Distribution of Motion Ranges Across Follow-Up Visits for NKI-RS Children

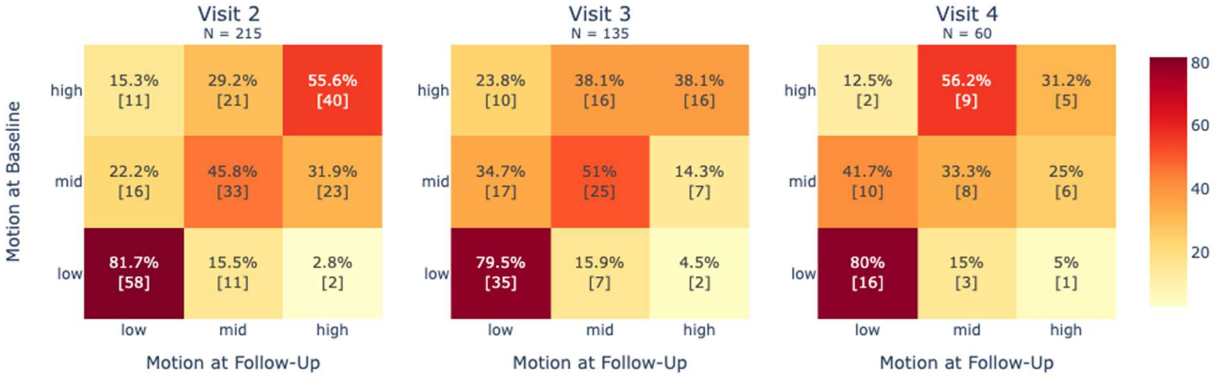

[A] All HBN and NKI-RS participants are represented by age. Red columns represent those with radiologist-reported motion artifacts while grey columns represent those without. Columns are overlapping. The line represents the percentage of participants with radiologist-reported motion artifacts (percentages are represented on the secondary y-axis on the right). The age distribution was significantly lower ($p < .001$) for participants with radiologist-reported motion artifacts. Rates of radiologist-reported motion artifacts decrease by age 12 (roughly coinciding with onset of puberty) and are negligible in adulthood.
[B] Euler number is represented for participants with and without radiologist-reported motion. Consistent with radiologist-reported artifacts, quantified motion artifacts are most prominent in children. However, quantified motion artifacts may be present in absence of clinically-reported motion.
[C] Motion terciles were defined based on the distribution of Euler numbers at baseline (e.g., the high motion tercile was defined as the top third of Euler numbers at baseline).

© 2024 Tobe RH et al. *JAMA Network Open.*

eFigure 8. Estimated Cost of Detecting Category 3 and Category 4 IFs in Clinical and Research Settings

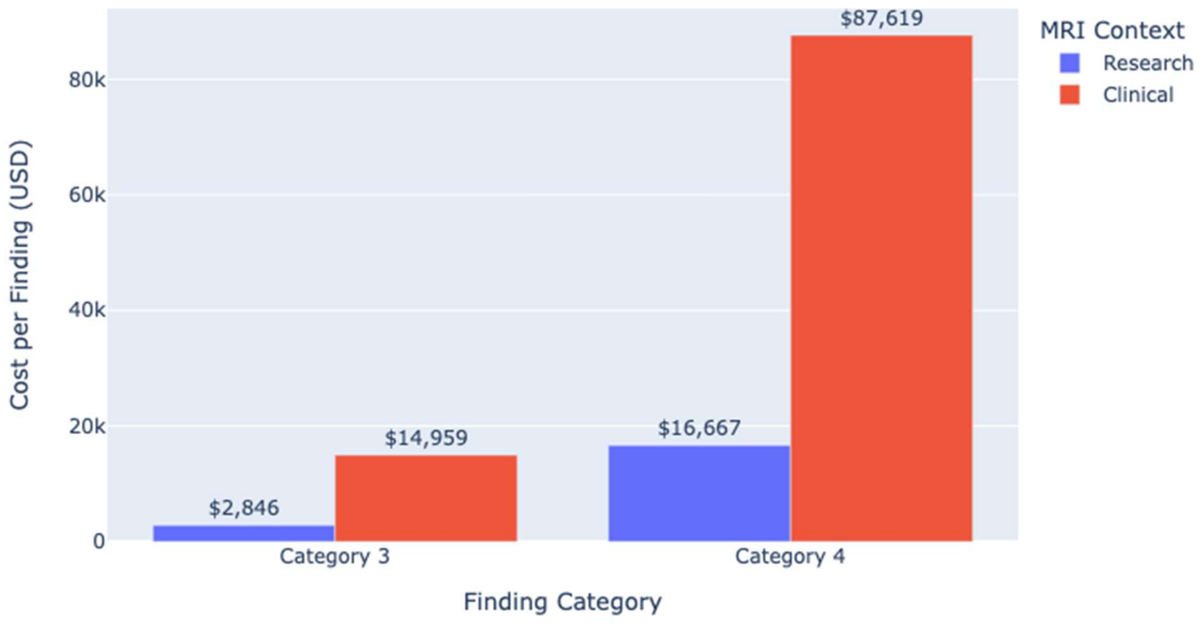

The approximate cost of detection for Category 4 findings was calculated as $87,619/finding for clinical settings and $16,667/finding for research settings.

© 2024 Tobe RH et al. *JAMA Network Open.*

References

1. Kaufman J, Birmaher B, Brent D, et al. Schedule for Affective Disorders and Schizophrenia for School-Age Children-Present and Lifetime Version (K-SADS-PL): initial reliability and validity data. *J Am Acad Child Adolesc Psychiatry*. 1997;36(7):980-988. doi:10.1097/00004583-199707000-00021
2. First M, Spitzer RL, Gibbon ML, Williams J. *Structured Clinical Interview for DSM-IV-TR Axis I Disorders, Research Version, Non-Patient Edition*.; 2002.
3. Achenbach TM, Rescorla LA. *Manual for the ASEBA School-Age Forms & Profiles*. University of Vermont, Research Center for Children, Youth, & Families; 2001.
4. Achenbach TM, Rescorla LA. *Manual for the ASEBA Adult Forms & Profiles*. University of Vermont, Research Center for Children, Youth, & Families; 2003.
5. Wechsler D. *Wechsler Abbreviated Scale of Intelligence, Second Edition (WASI-II)*.; 2011.
6. Smith PC, Schmidt SM, Allensworth-Davies D, Saitz R. A single-question screening test for drug use in primary care. *Arch Intern Med*. 2010;170(13):1155-1160. doi:10.1001/archinternmed.2010.140
7. WHO ASSIST Working Group. The Alcohol, Smoking and Substance Involvement Screening Test (ASSIST): development, reliability and feasibility. *Addiction*. 2002;97(9):1183-1194. doi:10.1046/j.1360-0443.2002.00185.x

© 2024 Tobe RH et al. *JAMA Network Open.*
