# Supplementary material for: Age, Motion, Medical, and Psychiatric Associations With Incidental Findings in Brain MRI
Source: JAMA Netw Open. 2024 Feb 13;7(2):e2355901. doi: 10.1001/jamanetworkopen.2023.55901 (PMC10865144; doi:10.1001/jamanetworkopen.2023.55901)
Supplement: Supplement 2. — Data Sharing Statement [file jamanetwopen-e2355901-s002.pdf]

Data Sharing Statement

Tobe. Age, Motion, Medical, and Psychiatric Associations With Incidental Findings in Brain MRI. *JAMA Netw Open*. Published February 13, 2024.
doi:10.1001/jamanetworkopen.2023.55901

Data
Data available: Yes
Data types: Deidentified participant data
How to access data: [NKI-RS] The primary website for all NKI-Rockland Initiatives, including the present, is located at: http://fcon_1000.projects.nitrc.org/indi/enhanced/index.html. Phenotypic data may be accessed through either an NKI-RS-dedicated instance of the Longitudinal Online Research and Imaging System (LORIS) located at https://data.rocklandsample.rfmh.org/ and supported by the NKI-RS research program staff or through the COINS Data Exchange (https://coins.trendscenter.org/). Except for age, sex, and handedness, which are publicly available with the imaging, NKI-RS phenotypic data are protected by a Data Usage Agreement (DUA). Investigators must complete the DUA (found at http://fcon_1000.projects.nitrc.org/indi/enhanced/data/DUA.pdf) and have it approved by an authorized institutional official before receiving access. The intent of the DUA is to ensure that data users (1) agree to protect participant confidentiality when handling the high dimensional NKI-RS phenotypic data (which includes single item responses) and (2) agree to take the necessary measures to prevent breaches of privacy. The DUA does not place any constraints on the range of analyses that can be carried out using shared data, nor does it include requirements for co-authorship by the originators of the NKI-RS. All imaging data can be accessed through the 1,000 Functional Connectomes Project and its International Neuroimaging Data-sharing Initiative (FCP/INDI) based at http://fcon_1000.projects.nitrc.org/indi/enhanced/neurodata.html. This website provides an easy-to-use interface with point-and-click download of datasets that have been previously compressed; it also provides directions for users who are interested in direct download of the data from an Amazon Simple Storage Service (S3) bucket. A CSV file with the complete list of S3 links is provided in the study website. However, if a more specific list of S3 links is required (e.g., sex = female & age <10), it can be generated though LORIS. Imaging data is stored in the Brain Imaging Data Structure (BIDS) format, which is an increasingly popular approach to describing MRI data in a standard format. All data are labeled with the participant's unique identifier. [HBN] Phenotypic data may be accessed through the COllaborative Informatics and Neuroimaging Suite (COINS) Data Exchange (http://coins.mrn.org/dx) or an HBN-dedicated instance of the Longitudinal Online Research and Imaging System (LORIS) located at http://data.healthybrainnetwork.org/. With the exception of age, sex and handedness, which are publicly available with the imaging, EEG and eye-tracking datasets, the HBN phenotypic data are protected by a Data Usage Agreement (DUA). Investigators must complete and have approved by an authorized institutional official before receiving access (the DUA can be found at: http://fcon_1000.projects.nitrc.org/indi/cmi_healthy_brain_network/sharing.html). Modeled after the practices of the NKI-Rockland Sample, the intent of the HBN DUA is to ensure that data users agree to protect participant confidentiality when handling the high dimensional HBN phenotypic data (which includes single item responses), and that they will agree to take the necessary measures to prevent breaches of privacy. With the exception of zip code (which is only available upon request), no protected health identifiers are present in data distributed through the DUA, as a means of ensuring minimal risk of privacy breach. The DUA does not place any constraints on the range of analyses that can be carried out using the shared data, nor does it include requirements for co-authorship by the originators of the HBN Biobank. All EEG, eye tracking and imaging data can be accessed through the 1,000 Functional Connectomes Project and its International Neuroimaging Data-sharing Initiative (FCP/INDI) based at http://fcon_1000.projects.nitrc.org/indi/cmi_healthy_brain_network. This website

provides an easy-to-use interface with point-and-click download of HBN datasets that have been previously compressed; the site also provides directions for those users who are interested in direct download of the data from an Amazon Simple Storage Service (S3) bucket. Imaging data is stored in the Brain Imaging Data Structure (BIDS) format, which is an increasingly popular approach to describing MRI data in a standard format. All data are labeled with the participant's unique identifier. EEG data are available openly, along with basic phenotypic data (age, sex, handedness, completion status of EEG paradigms) and performance measures for the EEG paradigms. These data are located in a comma-separated (.csv) file accessible via the HBN.
When available: With publication

Supporting Documents
Document types: None

Additional Information
Who can access the data: researchers who complete the Data Usage Agreement
Types of analyses: for any purpose
Mechanisms of data availability: with a signed Data Usage Agreement
